# MiR-19a-3p Suppresses M1 Macrophage Polarization by Inhibiting STAT1/IRF1 Pathway

**DOI:** 10.3389/fphar.2021.614044

**Published:** 2021-05-04

**Authors:** Xiaoxiao Zhu, Qiang Guo, Jing Zou, Bin Wang, Zhen Zhang, Ran Wei, Lin Zhao, Yunhong Zhang, Chu Chu, Xiaoxiao Fu, Xia Li

**Affiliations:** ^1^ Department of Obstetrics and Gynecology, The First Affiliated Hospital of Shandong First Medical University & Shandong Provincial Qianfoshan Hospital, Jinan, China; ^2^ School of Basic Medicine, Shandong First Medical University & Shandong Academy of Medical Sciences, Jinan, China; ^3^ Key Laboratory of Laparoscopic Technology, The First Affiliated Hospital of Shandong First Medical University, Jinan, China; ^4^ Department of Peripheral Vascular Disease, Affiliated Hospital of Shandong University of Traditional Chinese Medicine, Jinan, China

**Keywords:** M1 macrophage, macrophage polarization, miR-19a-3p, STAT1, post-transcriptional regulation

## Abstract

Macrophages, an important type of immune cells, are generally polarized to classically activated macrophage (M1) or alternatively activated macrophage (M2) to respond to environmental stimuli. Signal transducer and activator of transcription 1 (STAT1), a very important transcription factor, can promote M1 macrophage polarization. However, the mechanisms of regulating STAT1 in macrophage polarization remain unclear. In the present study, STAT1 was markedly elevated, however, miR-19a-3p was down-regulated in interferon (IFN)-γ and lipopolysaccharide (LPS) treated RAW264.7 cells, and dual-luciferase reporter assay identified that miR-19a-3p directly targeted STAT1 by binding to its 3′UTR. Up-regulated miR-19a-3p inhibited M1 polarization by targeting STAT1/interferon regulatory factor 1 (IRF1) and vice versa *in vitro*. Consistently, overexpression of miR-19a-3p in LPS treated mice by systemically administering agomiR-19a-3p effectively reduced the inflammation in mouse lung tissues, and inhibited M1 macrophage polarization via suppressing STAT1/IRF1 pathway. In summary, our study confirmed that miR-19a-3p, as a direct regulator of STAT1, inhibited M1 macrophages polarization. The miR-19a-3p/STAT1/IRF1 pathway can potentially be used to design novel immunotherapy for modulating macrophage polarization.

## Introduction

Macrophages, an essential component of innate immunity, have high plasticity and can display divergent phenotypes and functions (Lawrence and Natoli, 2011). In respond to different environmental stimuli, macrophages can develop into classically activated macrophages (M1 type) and alternatively activated macrophages (M2 type) (Franklin and Li, 2016; Cassetta and Pollard, 2020; Larionova et al., 2020b). M1 macrophages play a prominent role in immune surveillance by secreting pro-inflammatory cytokines and chemokines, and high antigen presentation. While M2 macrophages have significant effects in immune regulation by secreting cytokines IL-10 and/or TGF-β which are related to anti-inflammatory effect (Lawrence and Natoli, 2011; Hamilton et al., 2014; Bi et al., 2016; Shapouri-Moghaddam et al., 2018). Many transcription factors play important roles in macrophage polarization, such as interferon regulatory factors (IRFs), nuclear transcription factor-κB (NF-κB), signal transducer and activator of transcriptions (STATs), and so on (Lawrence and Natoli, 2011; Glass and Natoli, 2016; Huang et al., 2018; Li et al., 2018; Ma et al., 2018; Larionova et al., 2020a). Among them, STAT1 exerts a crucial role in modulating the polarization of macrophages.

STAT1, a major member of the STAT family, is activated by janus kinase (JAK) upon the stimulation of interferon (IFN)-γ (Osterlund et al., 2007). Activated STAT1 homodimers transfer into the nucleus and promote IFN-γ-response genes related to anti-virus, antigen present, and microbicidal function (Hu and Ivashkiv, 2009; Wynn et al., 2013). STAT1 is involved in cell differentiation, function and apoptosis processes, as well as tumor, chronic inflammation and autoimmune diseases (Li et al., 2015a; He et al., 2016; Dufour et al., 2018; Wang and Zhu, 2019). More recently, several researches uncovered the effects of STAT1 in macrophage polarization, and underline the significance of the STAT1-mediated macrophages’ activation (Kernbauer et al., 2012; Juhas et al., 2015). In human primary macrophage, RAW264.7 and THP-1 cells PARP14 silencing promoted IFN-γ-induced STAT1 activation and promoted pro-inflammatory macrophage polarization (Iwata et al., 2016). Ning Ding et al. pointed out that STAT1 played a significant role in M1 polarization, whereas physalin D suppressed the STAT1 activation to inhibit M1 polarization (Ding et al., 2019). These studies discover that STAT1 plays key roles in M1 polarization, while the up-stream factors that regulate STAT1 expression are poorly understood.

MicroRNAs (miRNAs) are highly conserved non-coding small RNAs, ranging from 21 to 25 nucleic acids. By binding to the 3′ untranslated region (3′UTR) of the target gene, miRNAs regulate the target gene at the post-transcriptional level (Iwakawa and Tomari, 2015; Xu et al., 2015). miRNAs are closely related to a series of important activities such as metabolism, cell cycle, growth, differentiation, proliferation, apoptosis, and so on (Urbich et al., 2008; Zhang, 2008). Emerging studies have indicated that miRNAs play important roles in macrophage polarization (Jaiswal et al., 2019; Miranda et al., 2018; Qian et al., 2020; Yu et al., 2017; Zhu et al., 2020). For instance, miR-1246 promotes M2 macrophage polarization by inhibiting telomeric repeat binding factor 2-interacting protein (TERF2IP) (Qian et al., 2020). MiR-145 is related to M2 macrophage polarization by inhibiting IL-16 and promoting IL-10 expression (Huang et al., 2019). MiR-99a suppresses M1 macrophage polarization via inhibiting tumor necrosis factor (TNF) expression (Jaiswal et al., 2019). Therefore, miRNAs have been shown to regulate macrophage polarization by strictly controlling transcription factors.

MiR-19a-3p, a member of the miR-17-92 miR cluster, is located on human chromosomes 13 and mouse chromosomes 14, respectively. A large number of studies have shown that miR-19a-3p is involved in the occurrence and development of a variety of cancers, including breast cancer, lung cancer, gastric cancer, hepatocellular carcinoma and so on (Wang et al., 2013a; Li et al., 2014; Lu et al., 2014; Li et al., 2015b; Tan et al., 2015; Ma et al., 2016; Feng et al., 2018; Wang et al., 2019), and high levels of serum miR-19a-3p can be used as an independent prognostic indicator in patients with non-small cell lung cancer and esophageal squamous cell carcinoma (Lin et al., 2013; Xu et al., 2014). Meanwhile, researches have reported miR-19a-3p is related to proliferation, apoptosis, metastasis, and chemo-resistance process of malignant tumors (Yang et al., 2014; Ma et al., 2016; Jiang et al., 2018; Wang et al., 2019). These studies indicate that miR-19a-3p is involved in a variety of physiological and pathological processes, but the role of miR-19a-3p in macrophage polarization has not been fully elucidated.

Recently, a growing number of researches have indicated that STAT1 can be controlled by some miRNAs. For example, up-regulated miR-146a can reduce NK cell-mediated cytotoxicity and the expression of TNF-α and IFN-γ by targeting STAT1 (Xu et al., 2017), and depresses T-cell immune function in chronic hepatitis B (CHB) patients by inhibiting STAT1 (Wang et al., 2013b). Moreover, enhancing the expression of miR-30a in subcutaneous fat pads of diabetic mice can increase insulin sensitivity, energy consumption, and reduce liver ectopic fat deposition and inflammation of white adipose tissue by inhibiting STAT1 signaling pathway (Koh et al., 2018). However, the mechanisms of miRNAs in macrophage polarization through targeting STAT1 are still unclear.

In this study, microarray analysis showed that miR-19a-3p was reduced, while STAT1 was increased significantly in M1 macrophages derived from RAW264.7 cells, and miR-19a-3p was negatively correlated with STAT1. Overexpression of miR-19a-3p suppressed M1 polarization whereas inhibition of miR-19a-3p showed the opposite result *in vitro.* Moreover, overexpressed miR-19a-3p reduce lung inflammation by depressing M1 macrophages in mice treated with LPS. Furthermore, we found that miR-19a-3p suppressed M1 polarization via inhibiting STAT1/IRF1 pathway by targeting STAT1. Our study confirmed that miR-19a-3p, as a direct regulator of STAT1, affected M1 macrophage polarization for the first time.

## Materials and Methods

### Cell Culture and Treatment

293T cells and RAW264.7 cells, purchased from the Cell Bank of Chinese Academy of Sciences (Shanghai, China), were cultured in DMEM (Biological Industries [BI], Beit Haemek, Israel) supplemented 10% FBS (BI) and 1% penicillin/streptomycin. Bone marrow-derived macrophages (BMDM) were derived from bone marrow cells of 7–9 weeks C57BL/6 mice. Bone marrow cells were flushed out through serum-free 1640 medium, and centrifuged at 1,500 rpm for 10 min. The cells were maintained in 1640 medium containing 10% FBS and 50 ng/mL M-CSF (Peprotech, Chicago, IL) for 7 days to establish mouse BMDM. All the cells were cultured at 37°C with 5% CO_2_. To obtain M1 macrophages, RAW264.7 cells and BMDM cells were stimulated with IFN-γ (Peprotech) at the concentration of 20 ng/ml and LPS (Sigma-Aldrich, St. Louis, MO, United States) at the concentration of 100 ng/ml for 24 h.

### Microarray Analysis

Mouse miRNA, release 21.0 (8*60K, Design ID: 070155) was used to detect miRNA expression in OE Biotech. Co., Ltd. (Shanghai, China). M1 macrophages derived from RAW264.7 cells (*n* = 3) and unstimulated RAW264.7 (*n* = 3) were used for microarray analysis. The sample treatment, data acquirement and analyzation were performed as previously described (Zhu et al., 2020). The microarray data have been deposited in Gene Expression Omnibus (GEO) database (https://www.ncbi.nlm.nih.gov/geo) (GSE143845).

### Cell Transfection

To study the regulatory effect of miR-19a-3p on STAT1, miR-19a-3p mimics, miR-19a-3p mimics negative control (NC), miR-19a-3p inhibitor, miR-19a-3p inhibitor NC (iNC) (GenePharma, Shanghai, China) were transfected into RAW264.7 cells with Lipofectamine 2000 (Invitrogen, Waltham, MA, United States) at a final oligonucleotide concentration of 100 nM for 24 h. Rescue experiment was performed in RAW264.7 cells by co-transfecting with miR-19a-3p mimics, STAT1 plasmid or NC, vector for 24 h via Lipofectamine 3000 (Invitrogen), followed with LPS plus IFN-γ treatment for 24 h. Table 1 shows the oligodeoxy nucleotide sequences used in the present study.

**TABLE 1:** miRNA sequences.

miRNAs	Sequences (5’-3’)
NC	Sense: UUC​UCC​GAA​CGU​GUC​ACG​UTT
	Antisense: ACG​UGA​CAC​GUU​CGG​AGA​AGA​ATT
miR-19a-3p mimics	Sense: UGU​GCA​AAU​CUA​UGC​AAA​ACU​GA
	Antisense: AGU​UUU​GCA​UAG​AUU​UGC​ACA​UU
iNC	CAG​UAC​UUU​UGU​GUA​GUA​CAA
miR-19a-3p inhibitor	UCA​GUU​UUG​CAU​AGA​UUU​GCA​CA

NC, mimics negative control; iNC, inhibitor negative control.

### RNA Isolation and Quantitative Real-Time PCR

RNA isolation from cells and mouse tissues and qRT-PCR were similar as our previous publication using commercial kits (Zhu et al., 2020). Briefly, Total RNA were purified from cells and tissues by TRIzol Reagent and converted to miRNA and mRNA cDNA by the miRNA 1st Strand cDNA Synthesis Kit (Vazyme, Nanjing, China) and the PrimeScript RT reagent Kit (Toyobo, Osaka, Japan) respectively. To amplify miRNA and mRNA, SYBR Green was used and qRT-PCR was performed on Applied Biosystems 7500 instrument. The amplification reaction for each sample was performed in triplicate. Relative quantification was calculated by the comparative 2^−ΔΔCt^ method. The primer sequences are displayed in Table 2.

**TABLE 2:** List of primers used in the study.

Gene	Forward primer (5’- 3’)	Reverse primer (5’- 3’)
CXCL9	CCG​AGG​CAC​GAT​CCA​CTA​CA	CCG​GAT​CTA​GGC​AGG​TTT​GA
CXCL10	GAT​GAC​GGG​CCA​GTG​AGA​AT	ATC​TCA​ACA​CGT​GGG​CAG​G
iNOS	AGC​CAA​GCC​CTC​ACC​TAC​TT	TCT​GCC​TAT​CCG​TCT​CGT​CC
TNF-α	ACG​GCA​TGG​ATC​TCA​AAG​AC	AGA​TAG​CAA​ATC​GGC​TGA​CG
STAT1	GTT​CCG​ACA​CCT​GCA​ACT​GAA	AGA​GGT​GGT​CTG​AAA​GGG​AAC
IRF1	AAA​GTC​CAA​GTC​CAG​CCG​AG	GTC​CGG​GCT​AAC​ATC​TCC​AC
ACTB	TCC​TTC​TTG​GGT​ATG​GAA​TCC​TG	TGC​TAG​GAG​CCA​GAG​CAG​TA
IRF4	AAC​TCC​GAC​AGT​GGT​TGA​TCG	CCC​TTC​TCG​GAA​CTT​GCC​TT
IRF7	GTG​ATC​TTT​CCC​AGT​CCT​GCT	TGC​CTA​CCT​CCC​AGT​ACA​CC
STAT3	ACG​AAA​GTC​AGG​TTG​CTG​GT	CAG​CAA​CAT​CCC​CAG​AGT​CTT
STAT6	CGG​AGC​TAC​TGG​TCA​GAT​CG	GGA​TGA​CGT​GTG​CAA​TGG​TG
Arginase-1	AAC​ACT​CCC​CTG​ACA​ACC​AG	CGC​AAG​CCA​ATG​TAC​ACG​AT
miR-19a-3p	TAA​TCA​CTG​TGC​AAA​TCT​ATG​CAA	TAT​GGT​TTT​GAC​GAC​TGT​GTG​AT
miR-19b-3p	TGT​CAT​AAT​CAC​TGT​GCA​AAT​CC	TAT​GGT​TTT​GAC​GAC​TGT​GTG​AT
miR-20a-5p	GCC​CGC​TAA​AGT​GCT​TAT​AGT​G	GCT​GTC​AAC​GAT​ACG​CTA​CGT
miR-20b-5p	CAA​AGT​GAT​CAT​AGT​GCA​GGT​A	GGG​ACC​TTG​GTT​AGG​TGC​AC
miR-18a-5p	ACGTAAGGTGCATCTAGTGVAGAT	GTGCAGGGTCCGAGGT
miR-93-5p	GCC​ATG​TAA​ACA​TCT​CGG​ACT​G	CAA​TGC​GTG​TGG​TGG​AGG​AG
U6	ATT​GGA​ACG​ATA​CAG​AGA​AGA​TT	GGA​ACG​CTT​CAC​GAA​TTT​G

CXCL9, chemokine (C-X-C motif) ligand 9; CXCL10, chemokine (C-X-C motif) ligand 10; iNOS, inducible NO synthase; TNF-α, tumor necrosis factor-α; STAT1, signal transducer and activator of transcription 1; IRF1, interferon regulatory factor 1; IRF4, interferon regulatory factor 4; IRF7, interferon regulatory factor 7; STAT3, signal transducer and activator of transcription 3; STAT6, signal transducer and activator of transcription 6; ACTB, beta-actin.

### Western Blot Analysis

Proteins from cells and tissues were extracted by RIPA buffer (Solarbio, Beijing, China) and quantified by BCA Protein Assay Kit (Thermo Scientific, Massachusetts). Antibodies against STAT1 (1:1,000, 14995, Cell Signaling Technology, Danvers, MA, United States), iNOS (1:1,000, 2982, Cell Signaling Technology, Danvers, MA, United States), phosphorylated STAT1 (p-STAT1, 1:1,000, 9167, Cell Signaling Technology), IRF1 (1:1,000, 8478, Cell Signaling Technology), Arginase-1 (1:1,000, 9819s, Cell Signaling Technology) and GAPDH (1:10,000, ab181603, Abcam, Cambridge, MA, United States) were used.

### Enzyme-Linked Immunosorbent Assay

The medium from RAW264.7 cells, IFN-γ plus LPS induced RAW264.7 cells, IFN-γ plus LPS induced RAW264.7 cells transfected with miR-19a-3p mimics or inhibitor, and mouse blood plasma sample were collected. TNF-α, Chemokine (C-X-C motif) ligand 9 (CXCL9), CXCL10 in the supernatants were detected with mouse TNF-α (70-EK282/3-96), CXCL9 (70-EK2143/2-24), CXCL10 (70-EK268/2-96) ELISA Kit (MultiSciences, Hangzhou, China). ELISA was performed on each sample in triplicate.

### Immunofluorescence

RAW264.7 cells were transfected with miR-19a-3p mimics, miR-19a-3p mimics NC, miR-19a-3p inhibitor, and miR-19a-3p iNC for 24 h respectively. After that, the cells were stimulated with LPS and IFN-γ for 24 h. Fixed by 4% paraformaldehyde for 30 min, the cells were infiltrated with 1% Triton X-100 for 10 min, and blocked in 10% normal mice serum for 30 min. After incubation with STAT1 (Cell Signaling Technology) or IRF1 antibody (Cell Signaling Technology) overnight at 4°C, FITC Mouse Anti-Rabbit IgG (BOSTER, Wuhan, China) was incubated for 1 h. The nuclei were counterstained using DAPI, and the sections were observed under fluorescence microscope (IX73, Olympus, Japan).

### Luciferase Reporter Assay

Dual luciferase reporter assay was carried out as previous described (Zhu et al., 2020). In brief, the wild-type (WT) or mutant (MUT) STAT1 3′UTR was cloned into pGL3-3M-Luc vector (Promega, Madison, WI). 293T cells were co-transfected with WT or MUT luciferase reporter plasmid and 100 nM mimics or NC of miR-19a-3p for 24 h. Luciferase activity in the cells was analyzed using the Dual-Luciferase® Reporter Assay System (Promega) on GloMax 20/20 Luminometer (Promega). Relative luciferase activity was standardized to the renilla luciferase luminescence.

### Flow Cytometric Analysis

Flow cytometric analysis was carried out as previous described (Zhu et al., 2020). In brief, RAW264.7 cells transfected with miR-19a-3p mimics or inhibitor followed by LPS plus IFN-γ treatment were collected. After washed with 1 × PBS for 2 times, cells were suspended in 100 ul 1 × PBS and stained with cluster of differentiation 80 (CD80, 553769, BD Biosciences, Franklin Lakes, NJ, United States), major histocompatibility complex Ⅱ (MHC Ⅱ, 12-5321-82, eBioscience, San Diego, CA, United States), cluster of differentiation 86 (CD86, 17-0862-81, eBioscience) antibody for 30 min at 4°C in the dark. Flow cytometry was carried out by cell quest software flow cytometer (BD Biosciences).

### Animal Experiments

Eight-week-old C57BL/6 mice were obtained from Beijing Biotechnology Co., Ltd., housed in the specific pathogen-free facility. The animal study was approved by the Animal Ethics Committee of Shandong First Medical University (No. SDFMU2018-A04) and performed in accordance with the National Academies Guiding Principles for the Care and Use of Laboratory Animals, 8th edition. Mouse (*n* = 10 per group) was injected with 100 μl saline or saline containing 10 nM miR-19a-3p agomiR (Ribobio, Guangzhou, China) or 10 nM agomiR NC (Ribobio) by tail vein for 1 h, and then treated with LPS (20 mg/kg) by intraperitoneal injection, as described in a previous study (Guo et al., 2020). After 24 h of LPS treatment, blood sampling in mice eyes were collected. After centrifugation at 3,000 rpm for 10 min, mouse plasma was collected and stored at –80°C for future experiments. Peritoneal macrophages were immediately collected as previously described (Ray and Dittel, 2010). In brief, mice were intraperitoneally injected with 5 ml ice-cold 1 × PBS containing 3% fetal bovine serum (FBS) followed by massage for 5 min. Peritoneal fluid was collected into 15 ml sterile centrifuge tube followed by centrifuging at 1,500 rpm/min for 8 min. Peritoneal macrophage were cultured in DMEM containing 10% FBS at 37°C with 5% CO_2_. Then, the supernatant was removed at 4 h, and the cells were washed with DMEM slowly to remove non-adherent cells. The purity of peritoneal macrophages was detected by flow cytometry using APC-conjugated antimouse F4/80 (17-4801-82, eBioscience) antibody. After collecting peritoneal macrophages, the lungs of mice were immediately collected.

### Hematoxylin and Eosin Staining

Lung tissues were fixed in 4% paraformaldehyde and made into 5 μm paraffin slices. Sections were then baked at 60°C for 1 h and dewaxed by xylene. After hydration, tissue sections were stained with H&E (Solarbio) following standard procedures, then dehydrated with alcohol gradients followed by clearing using xylene. All histological images were obtained by an optical microscope (E100; Nikon, Japan).

### Statistical Analysis

All experiments were performed at least three independent times unless otherwise stated. Values are listed as the mean ± SEM. The 2-tailed Student *t* test was applied to calculate the statistical significance between two different groups. One-way ANOVA followed by Bonferroni test were used for multiple comparisons. Correlations were analyzed by Pearson correlation. Data were plotted using GraphPad Prism 6.0 software (GraphPad Software, Inc., CA, United States). Statistical analyses were carried out by SPSS software (version 16.0). *p* < 0.05 was considered significant.

## Results

### STAT1 is Up-Regulated in M1 Macrophages

**FIGURE 1:**
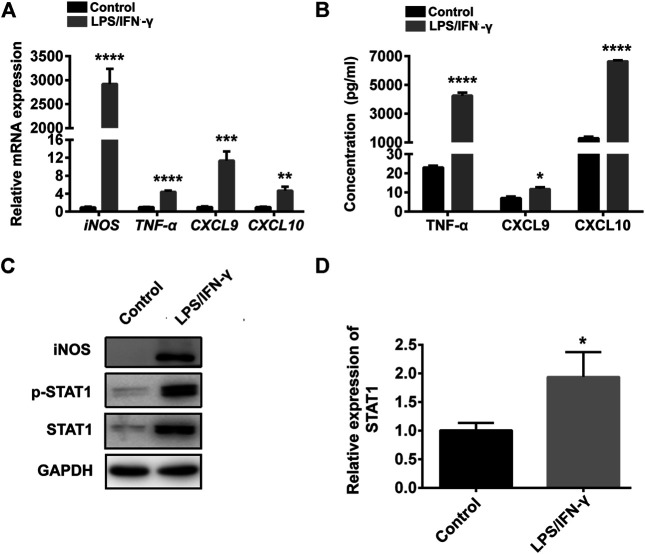
STAT1 was up-regulated in M1 macrophages. **(A)** CXCL9, CXCL10, iNOS, and TNF-α were determined by qRT-PCR in control cells (RAW264.7 cells) and RAW264.7 cells treated with LPS and IFN-γ (data were pooled from three independent experiments). **(B)** CXCL9, CXCL10, TNF-α were exhibited by ELISA in RAW264.7 cells treated with LPS and IFN-γ and control cells (data were pooled from three independent experiments). **(C)** STAT1, iNOS and p-STAT1 protein levels were measured in control cells and LPS plus IFN-γ induced RAW264.7 cells by western blot (a representative blot, from three independent experiments). **(D)** qRT-PCR analysis of STAT1 expression in control cells and LPS plus IFN-γ induced RAW264.7 cells (data were pooled from three independent experiments). Statistical significance was calculated using unpaired Student’s t-test. *p < 0.05, **p < 0.01, ***p < 0.001, ****p < 0.0001.

M1 macrophages were obtained by stimulating RAW264.7 cells with IFN-γ and LPS for 24 h. The mRNA and protein expression of CXCL9, CXCL10, iNOS, and TNF-α were obviously up-regulated in IFN-γ and LPS induced RAW264.7 cells (Figures 1A–C), indicating the RAW264.7 cells were polarized to M1 macrophages. Meanwhile, the mRNA, protein expression and phosphorylation level of STAT1 were notably elevated in IFN-γ and LPS induced RAW264.7 cells (Figures 1C,D), that were consistent with previous results (Iwata et al., 2016).

### MiR-19a-3p is Reduced in M1 Macrophages

**FIGURE 2:**
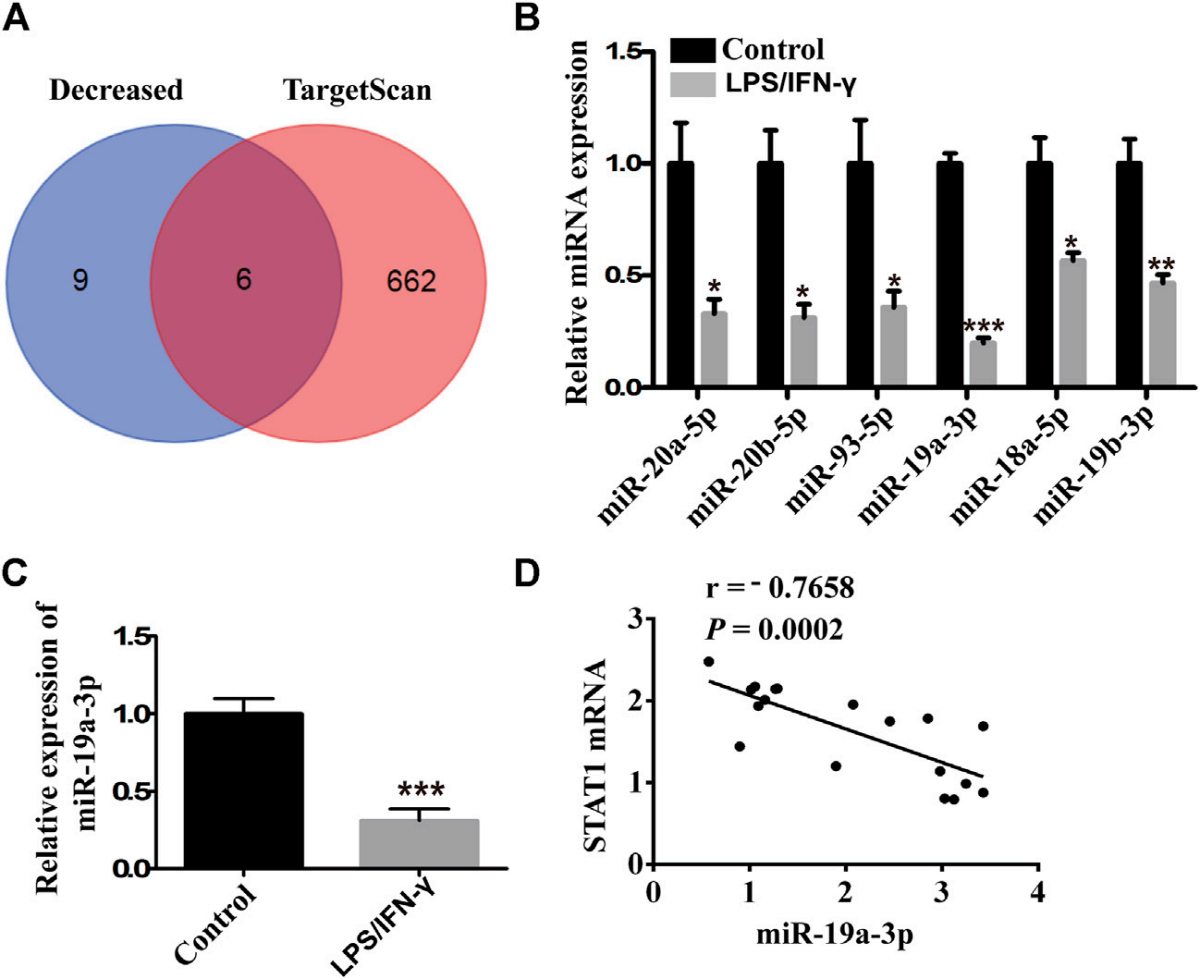
miR-19a-3p was decreased in M1 macrophages and negatively correlated with STAT1. **(A)** Schematic representation showed six potential miRNAs which might target STAT1 predicted by TargetScan in 15 most down-regulated miRNAs of microarray assay of RAW264.7 derived M1 macrophage. **(B)** miR-20a-5p, miR-20b-5p, miR-93-5p, miR-19a-3p, miR-18a-5p, miR-19b-3p were validated by qRT-PCR in the RAW264.7 cells treated with LPS and IFN-γ and control cells (RAW264.7 cells) of the miRNA microarray samples (data were pooled from three independent experiments with three samples per group). **(C)** miR-19a-3p in control cells (RAW264.7 cells) and LPS plus IFN-γ induced RAW264.7 cells was determined by qRT-PCR (data were pooled from three independent experiments). **(D)** Correlation between STAT1 mRNA and miR-19a-3p. Statistical significance was calculated using unpaired Student’s t-test. *p < 0.05, **p < 0.01, ***p < 0.001.

To find out the association of miRNA with STAT1 in M1 macrophages, three pairs of IFN-γ plus LPS treated RAW264.7 cells and untreated cells were used in miRNA microarray assay. There were 44 miRNAs which have six detected values were either up- or down-regulated at least 2-fold and *p* value < 0.05 in the IFN-γ and LPS stimulated RAW264.7 cells, including 17 increased miRNAs and 27 decreased miRNAs. To discover the decreased miRNAs which could target STAT1, TargetScan V7.2 was used for the prediction analysis in 15 most down-regulated miRNAs. Six miRNAs (miR-20a-5p, miR-20b-5p, miR-93-5p, miR-19a-3p, miR-18a-5p, miR-19b-3p) were predicted to bind with STAT1 mRNA 3′UTR (Figure 2A). While miR-19a-3p was the most stable down-regulated and selected for further analysis (Figures 2B,C). Moreover, miR-19a-3p was notably negative correlated with STAT1 (Figure 2D). These results suggested that down-regulated miR-19a-3p might promote M1 macrophages polarization by disinhibiting STAT1.

### MiR-19a-3p Influences M1 Macrophage Polarization

**FIGURE 3:**
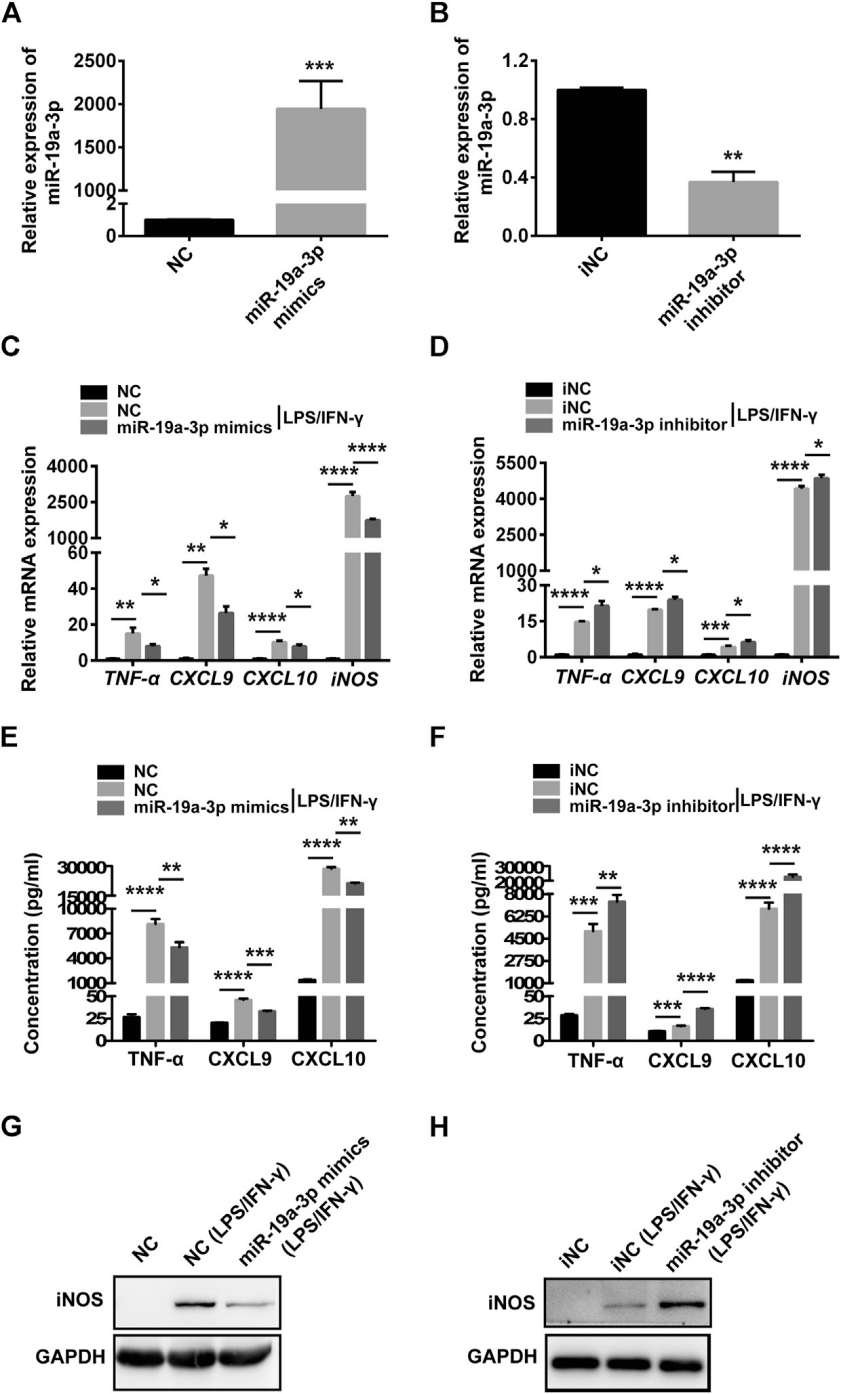
Overexpressed miR-19a-3p suppresses RAW264.7 derived M1 polarization and down-regulated miR-19a-3p improves RAW264.7 derived M1 polarization. RAW264.7 cells transfected with miR-19a-3p mimics/NC or miR-19a-3p inhibitor/iNC for 24 h, then stimulated with LPS plus IFN-γ for 24 h. **(A,B)** miR-19a-3p was determined using qRT-PCR (data were pooled from three independent experiments). **(C,D)** TNF-α, CXCL9, CXCL10, iNOS were analyzed by qRT-PCR (data were pooled from three independent experiments). **(E,F)** TNF-α, CXCL9, CXCL10 were exhibited by ELISA (data were pooled from three independent experiments). **(G,H)** Western blot analysis the protein level of iNOS (a representative blot, from three independent experiments). NC: miR-19a-3p mimics negative control, iNC: miR-19a-3p inhibitor negative control. Statistical significance was calculated using unpaired Student’s t-test or ANOVA followed by Bonferroni test. *p < 0.05, **p < 0.01, ***p < 0.001, ****p < 0.0001.

To explore the potential role of miR-19a-3p in M1 macrophage, miR-19a-3p mimics and inhibitor were used. The data showed that miR-19a-3p mimics up-regulated while inhibitor suppressed miR-19a-3p expression in RAW264.7 and BMDM cells (Figures 3A,B; Supplementary Figures S1A,B). IFN-γ plus LPS treatment obviously up-regulated M1 macrophage markers TNF-α, CXCL9, CXCL10, and iNOS mRNA expression, however, miR-19a-3p overexpression markedly decreased these markers (Figure 3C; Supplementary Figure S1C). In contrast, miR-19a-3p knockdown effectively enhanced the expression of these markers (Figure 3D; Supplementary Figure S1D). Meanwhile, the protein level of CXCL9, CXCL10, TNF-α, and iNOS were obviously down-regulated in miR-19a-3p over-expressed group (Figures 3E,G; Supplementary Figure S1E), and reducing the expression of miR-19a-3p showed the opposite effects (Figures 3F,H; Supplementary Figure S1F). Moreover, we found that M1 macrophage surface makers such as CD80, CD86 and MHC Ⅱ were significantly inhibited after miR-19a-3p mimics transfection (Supplementary Figure S2A). On the contrary, miR-19a-3p inhibitor has the opposite effect (Supplementary Figure S2B). In addition, we added experiments to observe the regulatory effect of miR-19a-3p on arginase-1. It was showed that LPS plus IFN-γ treatment inhibited the mRNA and protein expression of arginase-1, however, regulating the expression of miR-19a-3p had no effect on the mRNA and protein level of arginase-1 (Supplementary Figures S3A–D). All of these data suggest that miR-19a-3p can inhibit macrophage polarization to M1 phenotype.

### MiR-19a-3p Suppresses the Polarization of M1 Macrophage via STAT1/IRF1 Pathway

**FIGURE 4:**
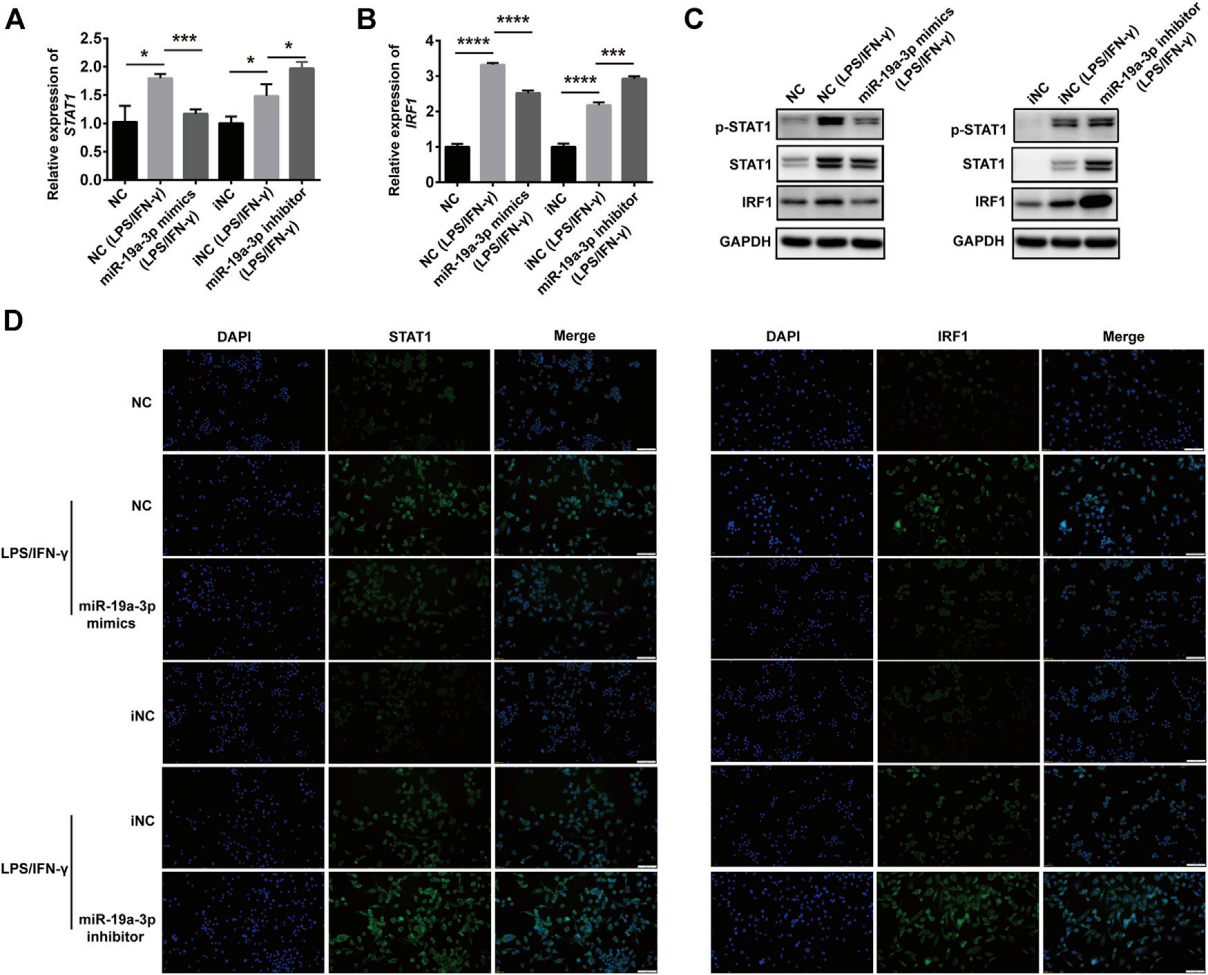
miR-19a-3p inhibits the STAT1/IRF1 pathway in RAW264.7 cells. RAW264.7 cells were transfected with miR-19a-3p mimics/NC or miR-19a-3p inhibitor/iNC for 24 h, then the cells were stimulated with LPS/IFN-γ for 24 h. **(A,B)** STAT1 and IRF1 mRNA expression were detected by qRT-PCR (data were pooled from three independent experiments). **(C)** p-STAT1, STAT1, and IRF1 were measured by western blot (a representative blot, from three independent experiments). **(D)** STAT1 and IRF1 were detected by immunofluorescence (a representative experiment, from three independent experiments). DAPI was used to stain the cell nucleus (Scale bar, 50 μm, 200×). NC: miR-19a-3p mimics negative control, iNC: miR-19a-3p inhibitor negative control. Statistical significance was calculated using ANOVA followed by Bonferroni test. *p < 0.05, ***p < 0.001, ****p < 0.0001.

To investigate whether miR-19a-3p affects macrophage polarization through STAT1/IRF1 pathway, we regulated the expression of miR-19a-3p by transfecting RAW264.7 and BMDM cells with miR-19a-3p mimics or inhibitor. We found that overexpression of miR-19a-3p decreased STAT1 and IRF1 mRNA expression efficiently, while down-regulated miR-19a-3p showed the opposite effects (Figures 4A,B; Supplementary Figures S4A,B). Meanwhile, up-regulated miR-19a-3p suppressed the protein level of p-STAT1, STAT1, and IRF1, while down-regulated miR-19a-3p enhanced these proteins expression (Figure 4C; Supplementary Figure S4C). Moreover, IF exhibited that overexpressed miR-19a-3p obviously inhibited the STAT1 and IRF1 expression, while down-regulated miR-19a-3p displayed an opposite result in IFN-γ plus LPS treated RAW264.7 (Figure 4D). Besides, the effect of miR-19a-3p on other transcription factor such as STAT3, STAT6, IRF4, IRF7 were also analyzed. The results showed that LPS plus IFN-γ treatment can inhibit IRF4, STAT6 expression while upregulate IRF7, STAT3 expression. However, regulating the expression of miR-19a-3p had no effect on the mRNA levels of STAT3, STAT6, IRF4 and IRF7 (Supplementary Figures S5A,B). These results proved that miR-19a-3p might suppress M1 macrophage phenotype polarization by inhibiting STAT1/IRF1 pathway.

**FIGURE 5:**
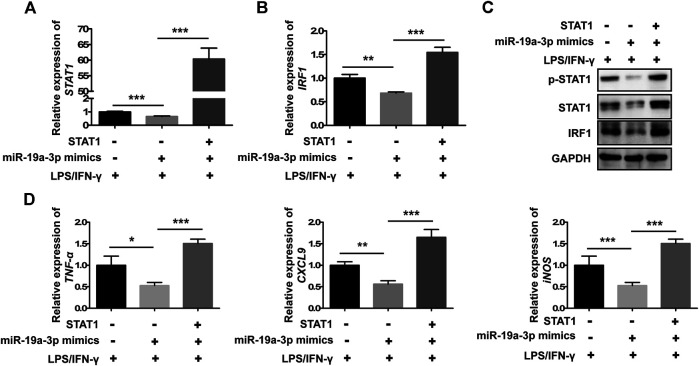
Overexpressed STAT1 reverses the inhibitory effect of miR-19a-3p in M1 phenotype polarization in RAW264.7 cells. RAW264.7 cells were co-transfected with miR-19a-3p mimics, STAT1 plasmid or NC, vector for 24 h, followed with LPS plus IFN-γ treatment for 24 h. **(A,B)** STAT1 and IRF1 mRNA levels were detected by qRT-PCR (data were pooled from three independent experiments). **(C)** p-STAT1, STAT1 and IRF1 protein levels were assessed by western blot (a representative blot, from three independent experiments). **(D)** TNF-α, CXCL9, and iNOS were detected by qRT-PCR (data were pooled from three independent experiments). NC: miR-19a-3p mimics negative control. Statistical significance was calculated using ANOVA followed by Bonferroni test. *p < 0.05, **p < 0.01, ***p < 0.001.

To further demonstrate whether miR-19a-3p inhibits the polarization of M1 macrophage through STAT1/IRF1 pathway, rescue experiment was performed. MiR-19a-3p mimics and STAT1 overexpressed plasmid were co-transfected into RAW264.7 cells followed with IFN-γ and LPS treatment. MiR-19a-3p mimics significantly inhibited the mRNA as well as the STAT1 phosphorylation and protein expression of STAT1, IRF1 (Figures 5A–C). While this effect was completely reversed by overexpressed STAT1 (Figures 5A–C). Furthermore, TNF-α, CXCL9 and iNOS were also reversed by overexpressed STAT1 (Figure 5D). These data showed that miR-19a-3p inhibited the polarization of M1 via suppressing STAT1/IRF1.

### STAT1 is a Direct Target Gene of miR-19a-3p

**FIGURE 6:**
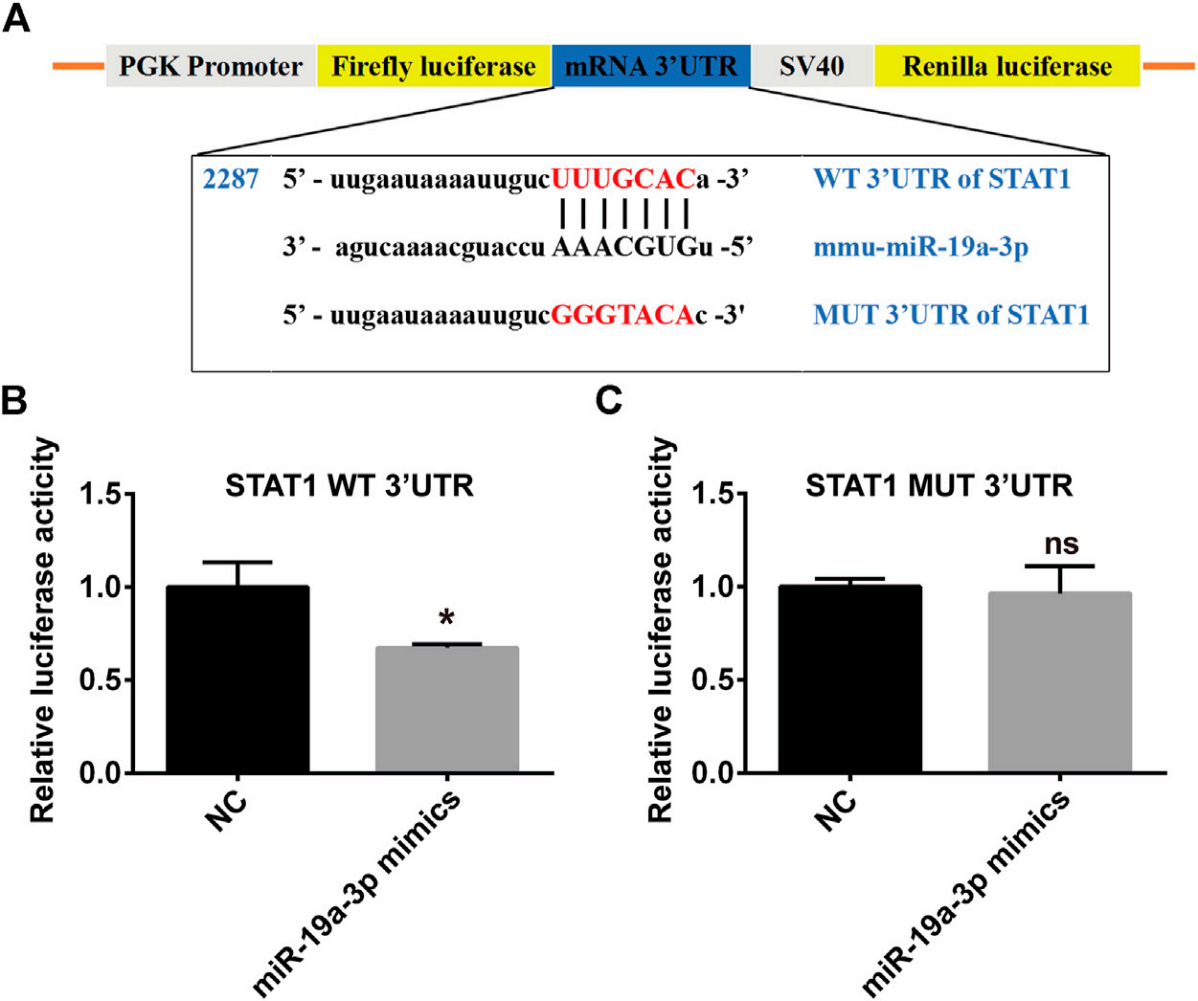
STAT1 is a direct target of miR-19a-3p. **(A)** Schematic representation of predicted binding sequence between the 3′UTR of STAT1 and miR-19a-3p, wild-type (WT) and mutant (MUT) STAT1 plasmid construction. **(B,C)** Transcriptional activity of STAT1 was determined by the dual-luciferase reporter assay in 293T cells (data were pooled from three independent experiments). Each sample had three replicates. Statistical significance was calculated using unpaired Student’s t-test. *p < 0.05, ns means no statistical difference.

To study the direct binding of miR-19a-3p with STAT1, plasmid with WT or MUT 3′UTR of STAT1 were constructed (Figure 6A), which was co-transfected with miR-19a-3p mimics or NC for dual-luciferase reporter assay. It was found that miR-19a-3p mimics significantly decreased luciferase reporter activity of WT 3′UTR plasmid of STAT1 (Figure 6B), but didn’t influence that of MUT 3′UTR plasmid (Figure 6C). These data above indicated that STAT1 was a direct target gene of miR-19a-3p.

### Overexpressed miR-19a-3p Inhibits M1 Polarization in LPS-Treated Mice

**FIGURE 7:**
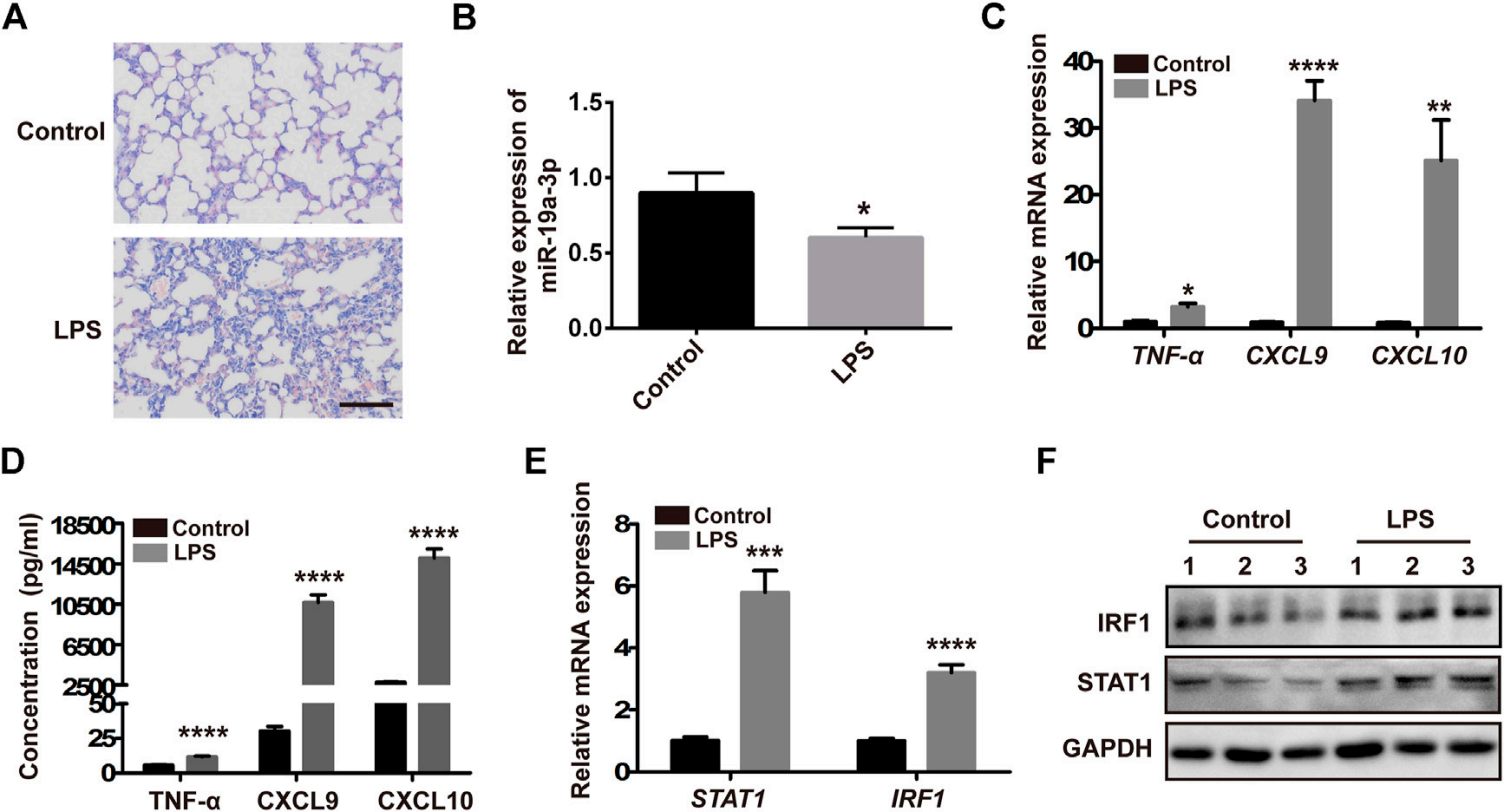
M1 macrophage increased obviously in the LPS challenged sepsis model. Male 5C7BL/6 J mice were challenged with 20 mg/kg LPS or saline by intraperitoneal injection for 24 h. **(A)** H&E staining of lung tissues treated with LPS and control mice (a representative experiment, from three independent experiments) (Scale bar, 50 μm, 200×). **(B,C)** miR-19a-3p, TNF-α, CXCL9, and CXCL10 in the peritoneal macrophages of mice treated with LPS and control mice were determined using qRT-PCR (data were pooled from three independent experiments with six mice per group). **(D)** TNF-α, CXCL9, and CXCL10 in serum of mice treated with LPS and control mice were measured by ELISA (data were pooled from three independent experiments with six mice per group). **(E)** STAT1 and IRF1 were detected in the peritoneal macrophages of mice treated with LPS and control mice (data were pooled from three independent experiments with six mice per group). **(F)** IRF1 and STAT1 were measured by western blot in the peritoneal macrophages of mice treated with LPS and control mice (a representative blot, from three independent experiment with three mice per group). Statistical significance was calculated using unpaired Student’s t-test. *p < 0.05, **p < 0.01, ***p < 0.001, ****p < 0.0001.

To research the precise effect of miR-19a-3p in M1 macrophage polarization *in vivo*, a mouse sepsis model was used. Male C57BL/6 J mice were treated with 20 mg/kg LPS or saline by intraperitoneal injection, after 24 h, we found that challenged with LPS increased the infiltration of inflammatory cells obviously in lung tissues compared with control mice (Figure 7A). MiR-19a-3p was down-regulated in the peritoneal macrophages of mice that challenged with LPS (Figure 7B). Meanwhile, TNF-α, CXCL9, CXCL10 were increased in the peritoneal macrophages after treated with LPS (Figures 7C,D). In addition, LPS treated up-regulated the expression of STAT1 and IRF1 (Figures 7E,F). These results suggested that M1 macrophage increased obviously in the LPS challenged sepsis model, and miR-19a-3p regulated STAT1/IRF1 signaling pathway is involved in this process.

**FIGURE 8:**
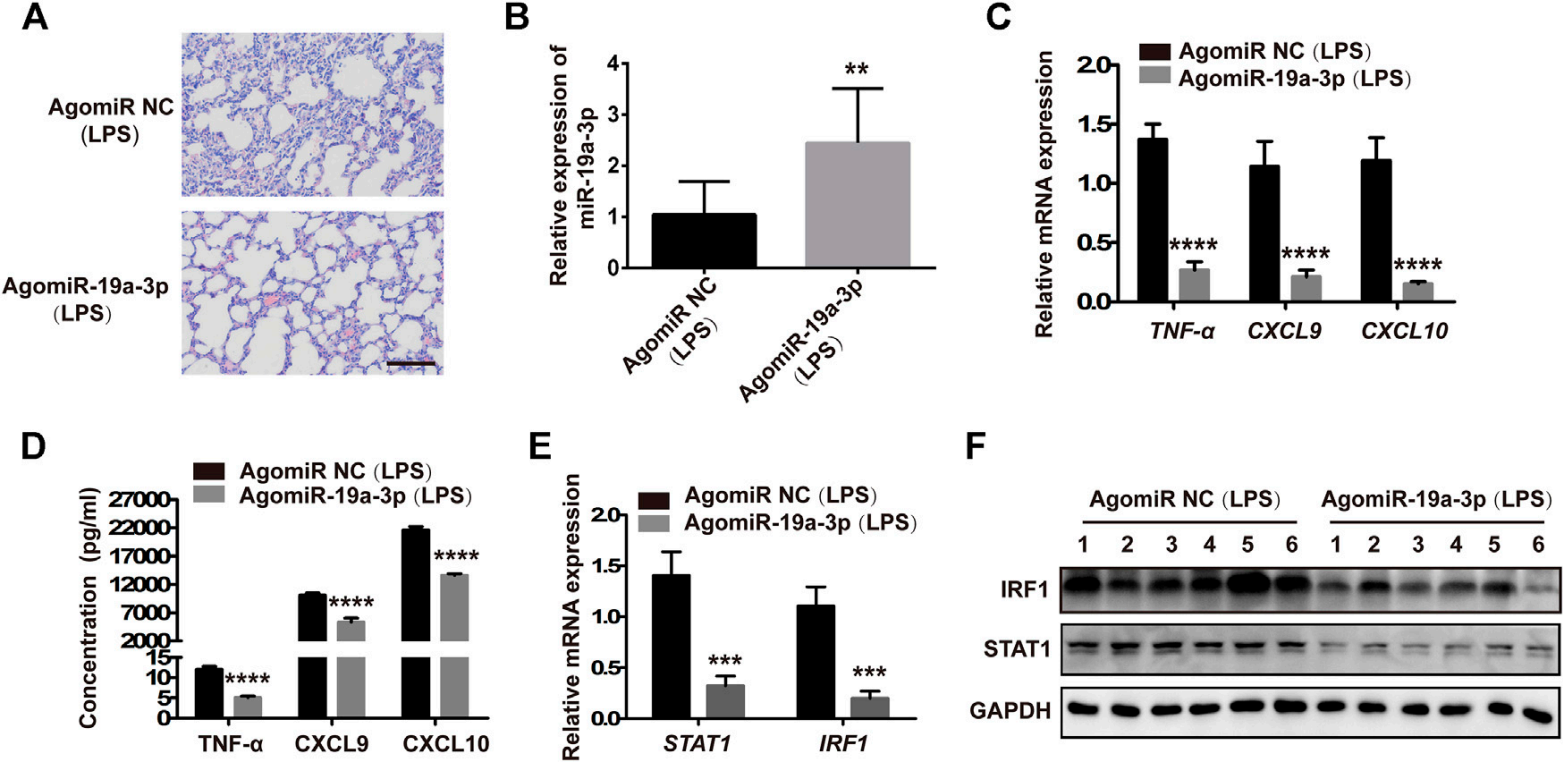
Overexpressed miR-19a-3p reduced the inflammation of lung tissues and inhibited M1 polarization in LPS-treated mice. Male 5C7BL/6 J mice were treated with agomiR-19a-3p or agomiR NC for 1 h, followed by the 20 mg/kg LPS challenge by intraperitoneal injection. **(A)** H&E staining of lung tissues of mice treated with agomiR-19a-3p or agomiR NC followed by challenging LPS (a representative experiment, from three independent experiments). (Scale bar, 50 μm, 200×). **(B,C)** miR-19a-3p, TNF-α, CXCL9, and CXCL10 in the peritoneal macrophages of mice using qRT-PCR (data were pooled from three independent experiments with ten mice per group). **(D)** TNF-α, CXCL9, and CXCL10 in serum of mice were measured by ELISA (data were pooled from three independent experiments with ten mice per group). **(E)** STAT1 and IRF1 were detected in the peritoneal macrophages of mice (data were pooled from three independent experiments with 10 mice per group). **(F)** IRF1 and STAT1 were measured by western blot in the peritoneal macrophages of mice (a representative blot, from three independent experiment with six mice per group). Statistical significance was calculated using unpaired Student’s t-test. **p < 0.01, ***p < 0.001, ****p < 0.0001.

To confirm that the STAT1/IRF1 signal pathway regulated by miR-19a-3p plays an important role in M1 macrophage polarization in LPS challenged sepsis model. Male C57BL/6 J mice were administered with 10 nM agomiR-19a-3p or agomiR NC by tail vein injection, and then treated with 20 mg/kg LPS by intraperitoneal injection. The pre-treatment of agomiR-19a-3p significantly reduced the infiltration of inflammatory cells in the lung tissues compared with that in agomiR NC groups (Figure 8A). MiR-19a-3p was increased in the peritoneal macrophages of the agomiR-19a-3p group (Figure 8B), in which TNF-α, CXCL9, CXCL10 decreased obviously compared with that in agomiR NC group (Figures 8C,D). Meanwhile, STAT1 and IRF1 were obviously reduced in agomiR-19a-3p group (Figures 8E,F). These data proved that miR-19a-3p could inhibit M1 macrophage polarization by STAT1/IRF1 pathway *in vivo*.

## Discussion

Macrophages, an essential component of innate immunity, present throughout all tissues and have high plasticity in respond to different environmental stimuli (Wynn et al., 2013; Bassler et al., 2019). Studies have reported that macrophage are associated with health and pathological conditions, such as metabolic homeostasis, immunity, chronic inflammatory, autoimmune diseases (Aaronson and Horvath, 2002; Murray, 2017; Dufour et al., 2018). Modulating the polarization of macrophages contributes to eliminate inflammation in the progression of chronic inflammation and autoimmune diseases (Dufour et al., 2018). However, the potential mechanisms of macrophage polarization remain unclear. In this study, miR-19a-3p reduced while STAT1 increased significantly in M1 macrophages, and miR-19a-3p was negatively correlated with STAT1. Overexpressed miR-19a-3p suppressed M1 polarization whereas decreased miR-19a-3p exhibited the opposite result *in vitro.* Moreover, overexpressed miR-19a-3p reduced lung inflammation and inhibited M1 polarization in LPS-treated mice *in vivo*. Furthermore, miR-19a-3p inhibited M1 polarization by suppressing STAT1/IRF1 pathway. This study presented that miR-19a-3p could regulate the polarization of M1 macrophage by inhibiting STAT1/IRF1 pathway.

Increasing evidence has shown that the polarization of macrophages is regulated by a few transcription factors (Juhas et al., 2015; Glass and Natoli, 2016; Huang et al., 2018; Li et al., 2018; Larionova et al., 2020a). Among these transcription factors, STAT1 plays key roles in modulating macrophage polarization. STAT1 belongs to the STATs family and is a prototypical member of the JAK/STAT pathway (Aaronson and Horvath, 2002). STAT1 signaling pathway is involved in the cross-talk between innate and adaptive immunity (Hu and Ivashkiv, 2009; Sikorski et al., 2012; Chmielewski et al., 2014). It was reported that ferric ammonium citrate treatment could inhibit RAW264.7-derived M1 macrophage by suppressing STAT1 (Gan et al., 2017). Chrissy M. Leopold Wager et al. indicated that STAT1 played key roles in M1 macrophage polarization and NO production during Cryptococcus neoformans infection (Leopold Wager et al., 2015). In our study, it was found that both total and phosphorylated STAT1 were obviously increased in IFN-γ and LPS induced M1 macrophages that were consistent with previous results (Iwata et al., 2016). However, the upstream mechanisms of regulating STAT1 in macrophage polarization remain unclear.

**FIGURE 9:**
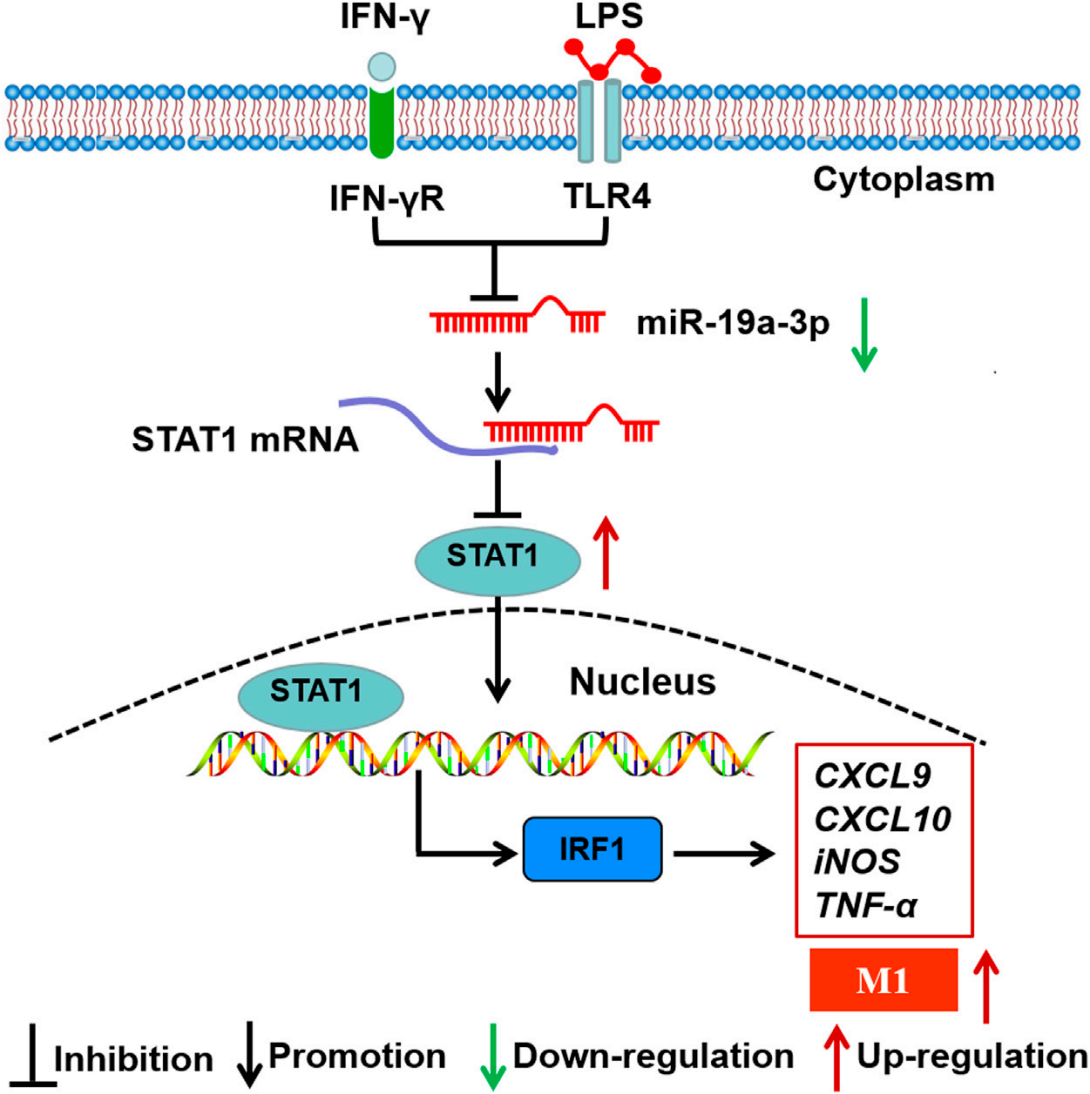
Schematic representation of the role of miR-19a-3p in regulating M1 macrophage polarization. MiR-19a-3p was down-regulated in M1 macrophage, and decreased miR-19a-3p promoted M1 macrophage polarization through STAT1/IRF1 pathway. MiR-19a-3p decreased STAT1 expression by directly targeting its mRNA 3′UTR and then inhibited IRF1.

Recently, accumulating studies demonstrate that miRNAs play crucial roles in M1 macrophage polarization, such as miR-1246, miR-145, miR-146a (Huang et al., 2016; Huang et al., 2019; Jaiswal et al., 2019; Qian et al., 2020). Meanwhile, accumulating researches have demonstrated that STAT1 is regulated by some miRNAs. For instance, miR-944 inhibits lung adenocarcinoma tumorigenesis by suppressing STAT1 (An et al., 2019). miR-499a inhibits LPS induced inflammation and apoptosis in human vascular endothelial cells by targeting STAT1 (Wang and Zhu, 2019). Although studies have found that miRNAs regulate STAT1 expression and M1 macrophage polarization, the roles and underlying mechanisms of miRNAs in regulating macrophage polarization through targeting STAT1 are still unclear. In this study, we found that miR-19a-3p was obviously down-regulated in IFN-γ and LPS induced RAW264.7 cells, and negatively correlated with STAT1. These data indicated that miR-19a-3p might affect M1 macrophage polarization by regulating STAT1 expression. As expected, overexpressed miR-19a-3p suppressed M1 markers TNF-α, CXCL9, CXCL10, iNOS, CD80, CD86, and MHCⅡ expression, while down-regulated miR-19a-3p showed opposite results. Meanwhile, up-regulated miR-19a-3p reduced the expression of STAT1 and IRF1 and vice versa *in vitro*. More importantly, overexpressed STAT1 reversed the inhibitory effect of miR-19a-3p in M1 polarization and IRF1 expression. Dual-luciferase reporter assay confirmed the binding site between miR-19a-3p and STAT1. These data proved that miR-19a-3p could suppress M1 polarization by inhibiting STAT1/IRF1 pathway. Consistent with the above results, our study *in vivo* showed that overexpressed miR-19a-3p in LPS treated mice effectively reduced the inflammation in mouse lung tissues, suppressed M1 macrophage polarization and inhibited STAT1, IRF1 expression. These results further demonstrated miR-19a-3p inhibit M1 macrophage polarization by suppressing STAT1/IRF1 pathway. Our finding was different from Yang et al., results that miR-19a-3p can inhibit the M2 phenotype polarization of tumor associated macrophages by regulating Fra-1 (Yang et al., 2014). The reasons for the different findings may be as follows. Firstly, it should be noticed that the expression profiles of miRNAs and genes expressed differently in different induction conditions. Secondly, the underlying mechanisms of macrophage polarization regulated by miRNAs are diverse, which might involve various target genes and cross-talk among miRNAs. Therefore, in different induced conditions, miR-19a-3p may regulate different genes and then produce different regulatory effects. In summary, the present study reveals for the first time that overexpression of miR-19a-3p can suppress M1 macrophage polarization both *in vitro* and *in vivo*. MiR-19a-3p downregulates STAT1 expression by targeting STAT1 3′UTR, thus inhibits IRF1 expression and M1 macrophage phenotype polarization (Figure 9). The miR-19a-3p/STAT1/IRF1 axis may be a potential target in macrophage polarization, and highlights a better comprehension for potential mechanism of pathogenesis and progression of diseases such as chronic inflammatory and autoimmune diseases. Our findings may provide promising biomarkers for prognosis of cancer patients, as well as therapeutic targets to design novel immunotherapy for the treatment of cancers including lung cancer. Meanwhile, it should be noticed that since the regulation of macrophage polarization and function is complicated, the role of miR-19a-3p on macrophage still needs further in-depth research.

## Data Availability

The raw data supporting the conclusions of this article will be made available by the authors, without undue reservation. The datasets presented in this study can be found in NCBI using the accession GSE143845.
