# Supplementary figures and images for: MiR-19a-3p Suppresses M1 Macrophage Polarization by Inhibiting STAT1/IRF1 Pathway
Source: Front Pharmacol. 2021 May 4;12:614044. doi: 10.3389/fphar.2021.614044 (PMC8129022; doi:10.3389/fphar.2021.614044)

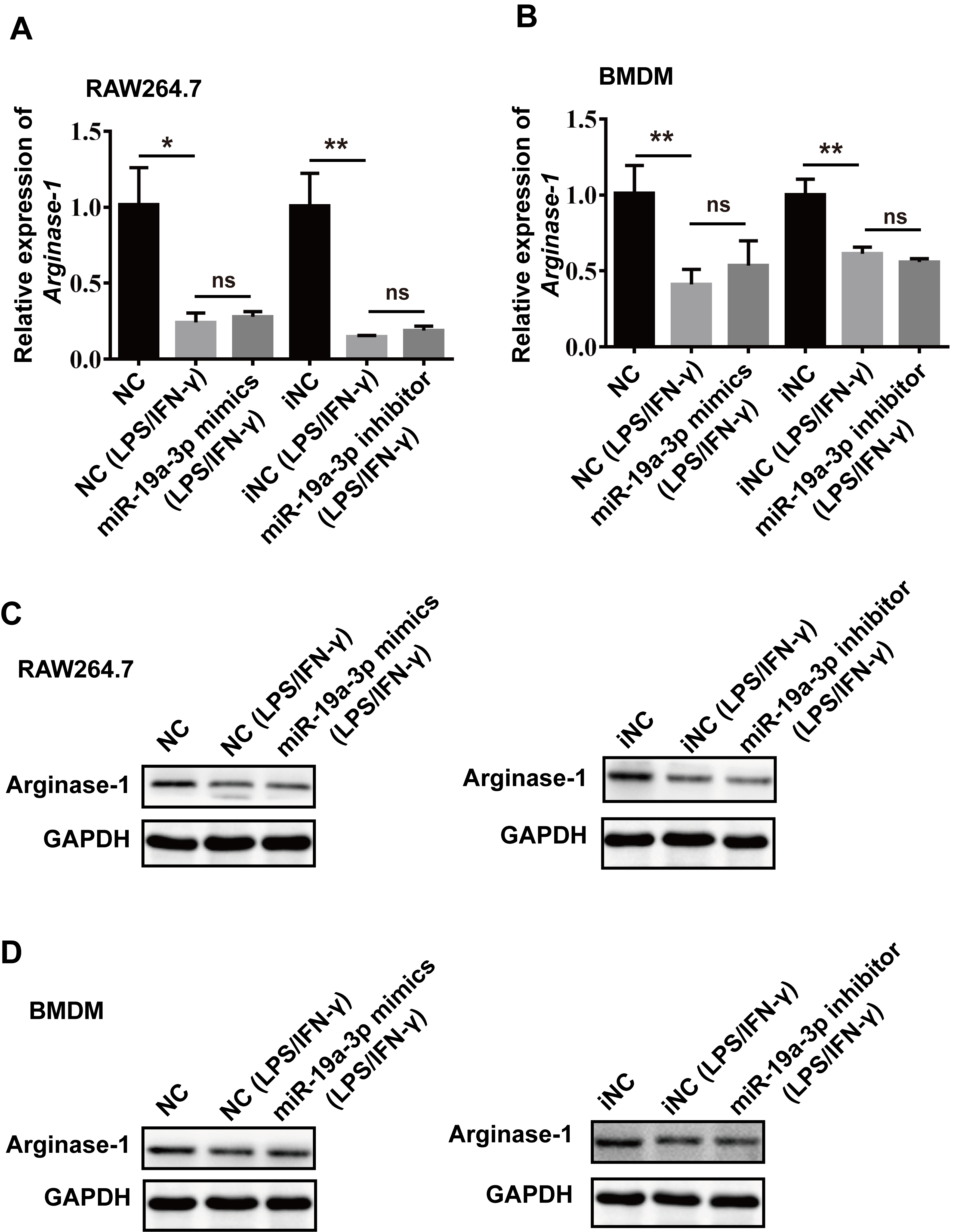

Supplement: Supplementary file 1 [file image3.tif]

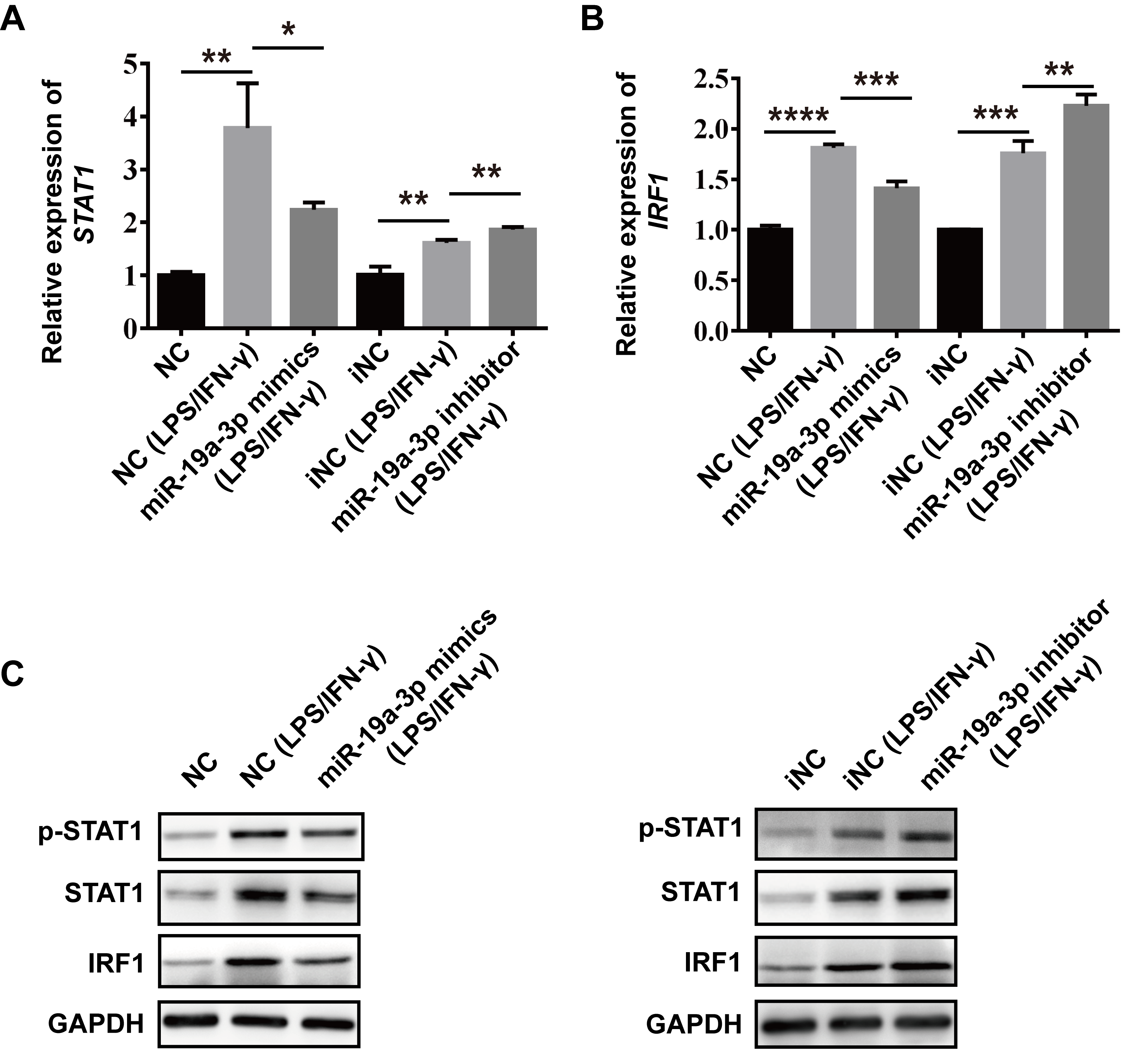

Supplement: Supplementary file 2 [file image4.tif]

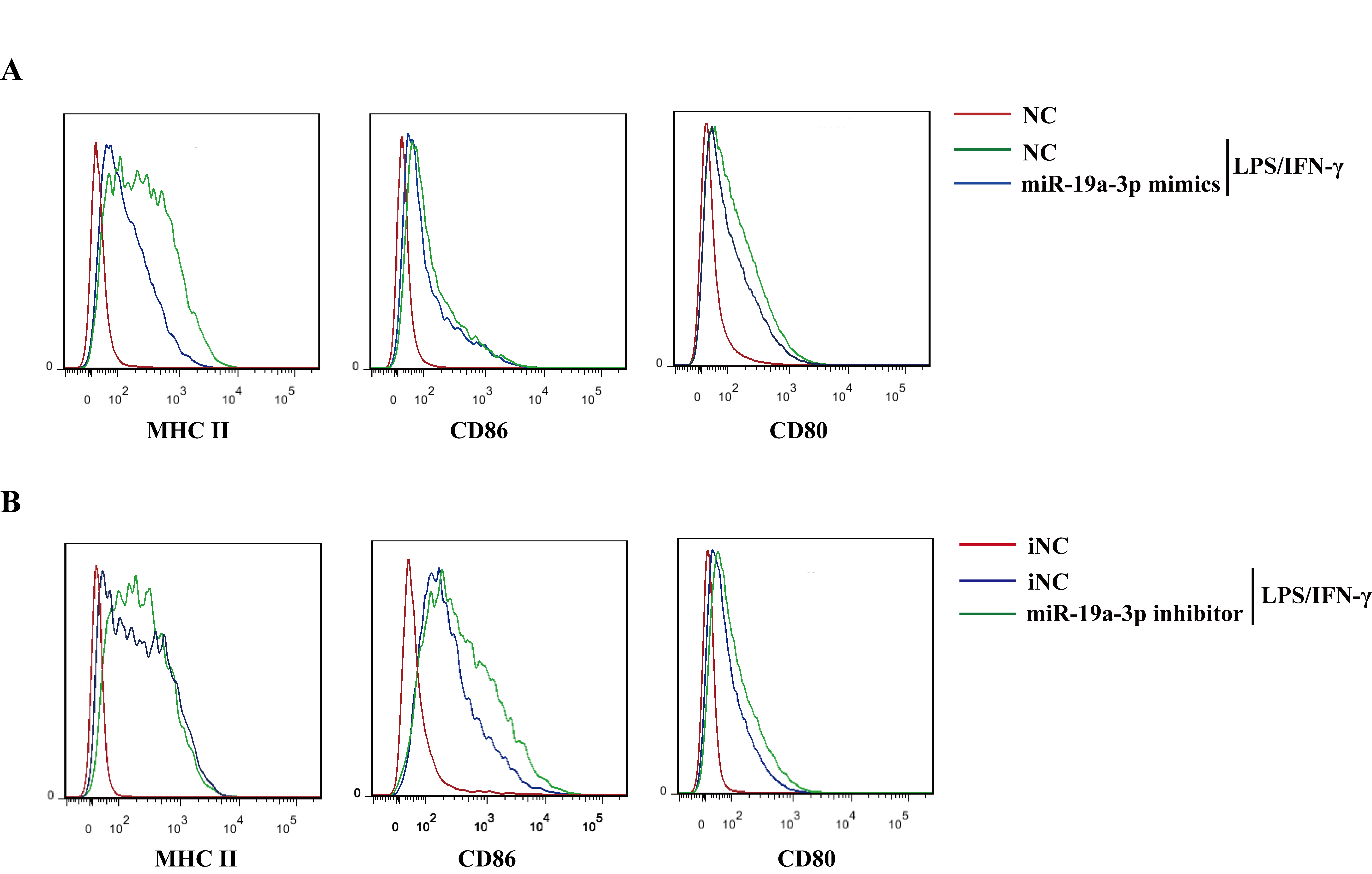

Supplement: Supplementary file 4 [file image2.tif]

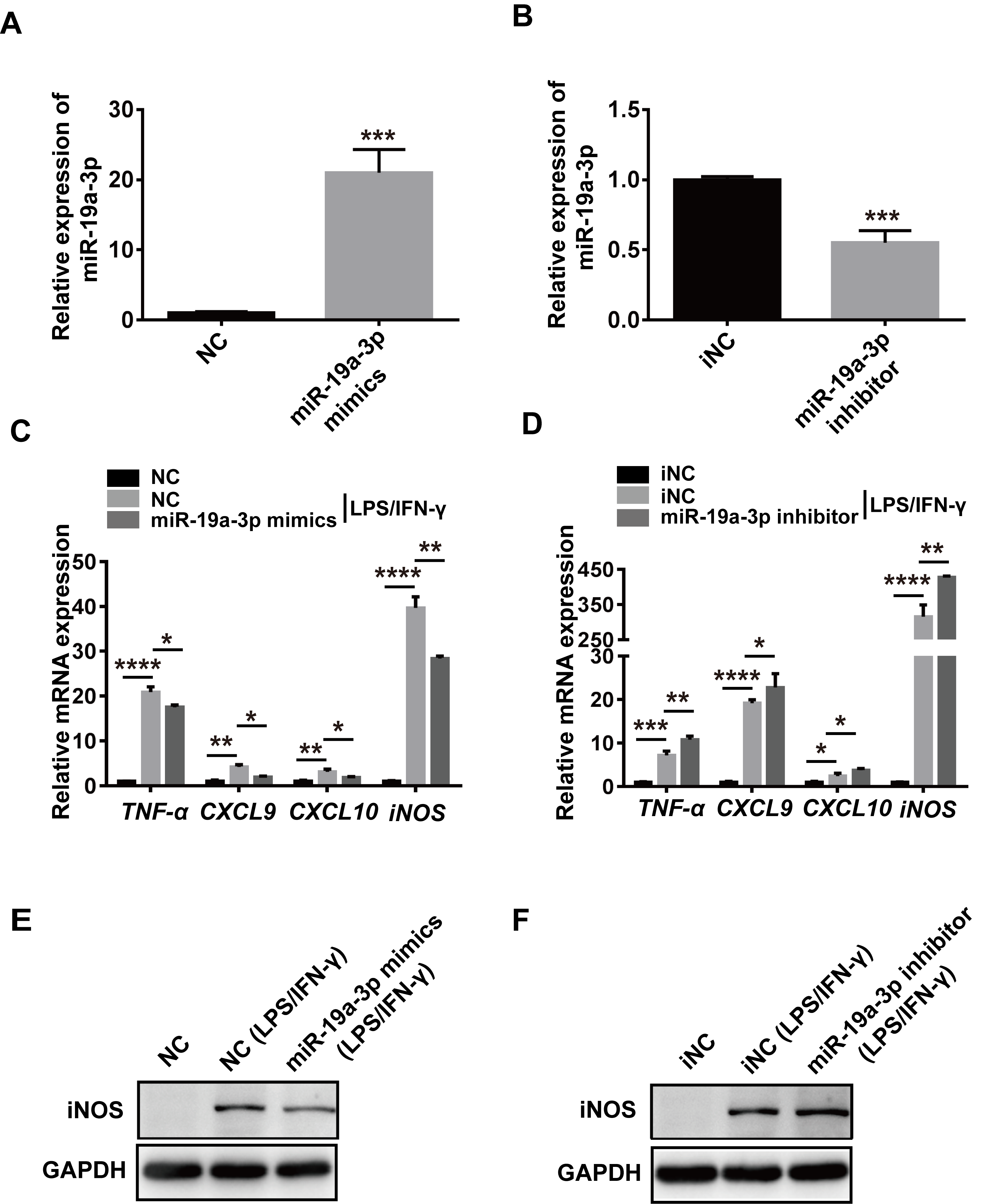

Supplement: Supplementary file 5 [file image1.tif]

-----

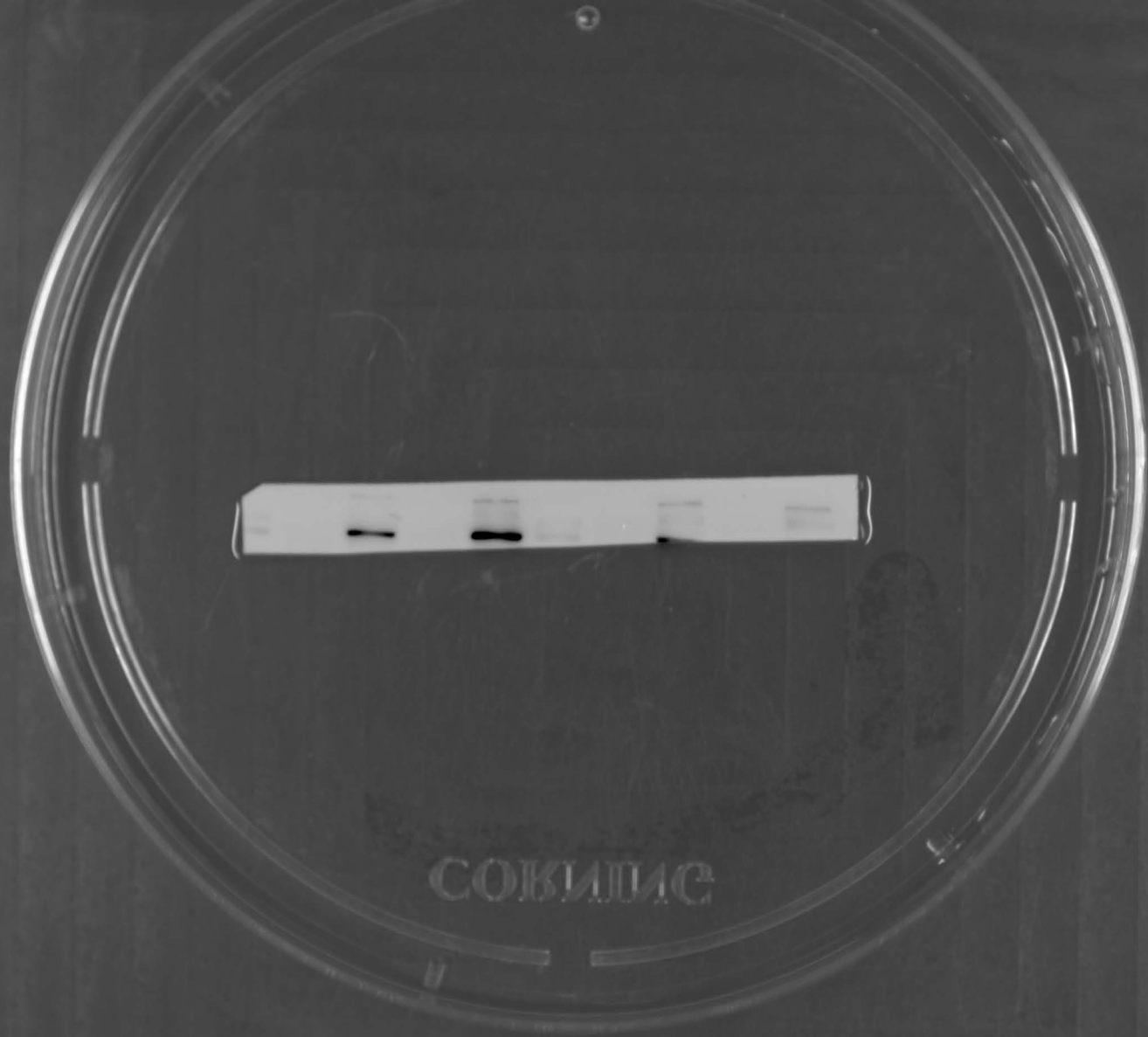

COVILIC

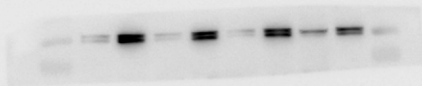

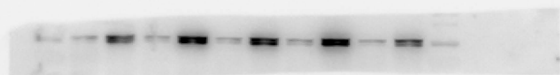

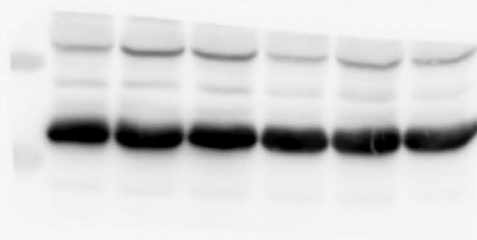

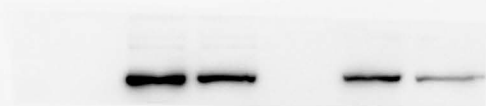

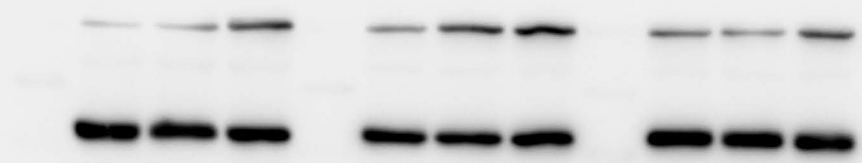

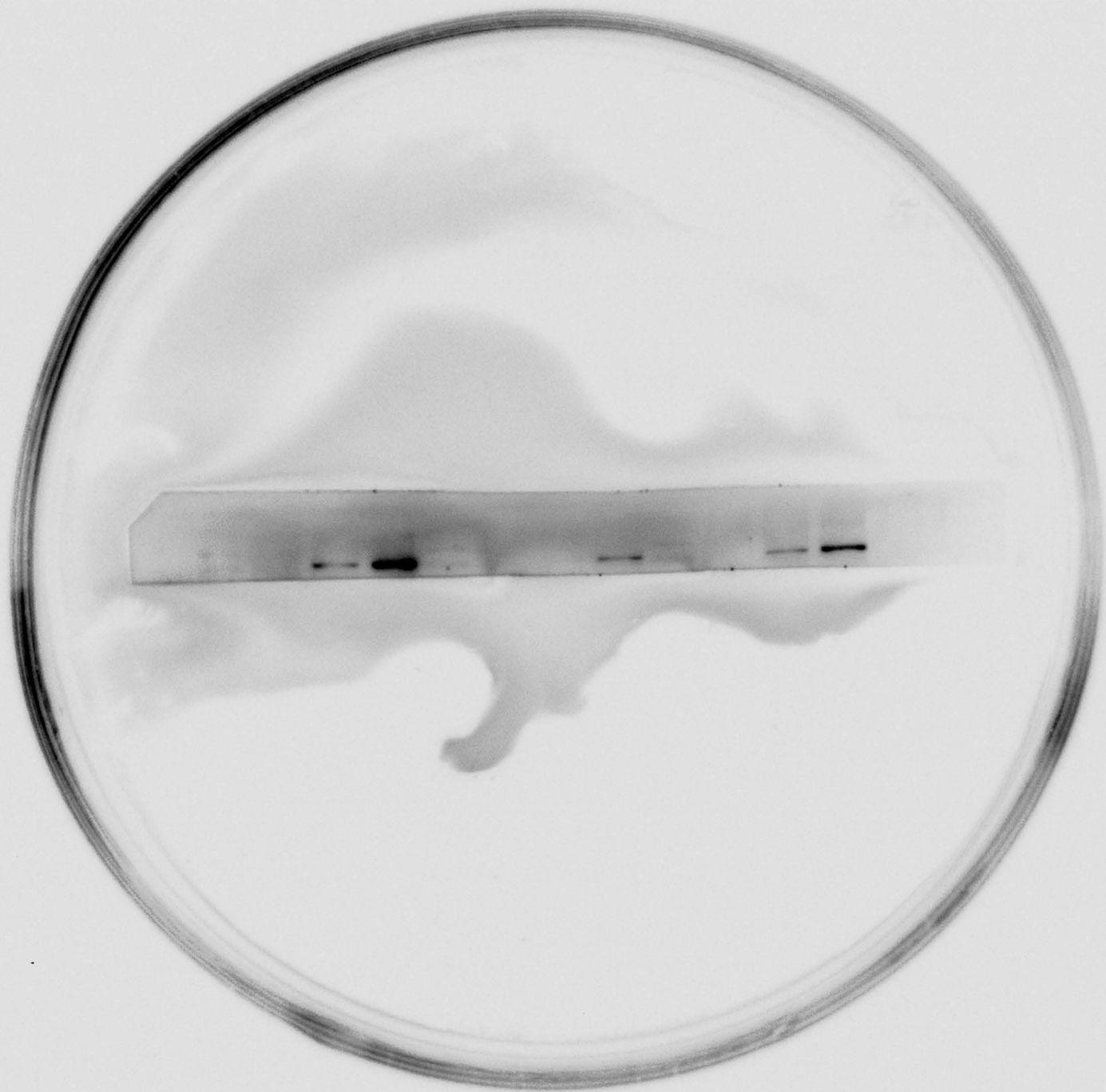

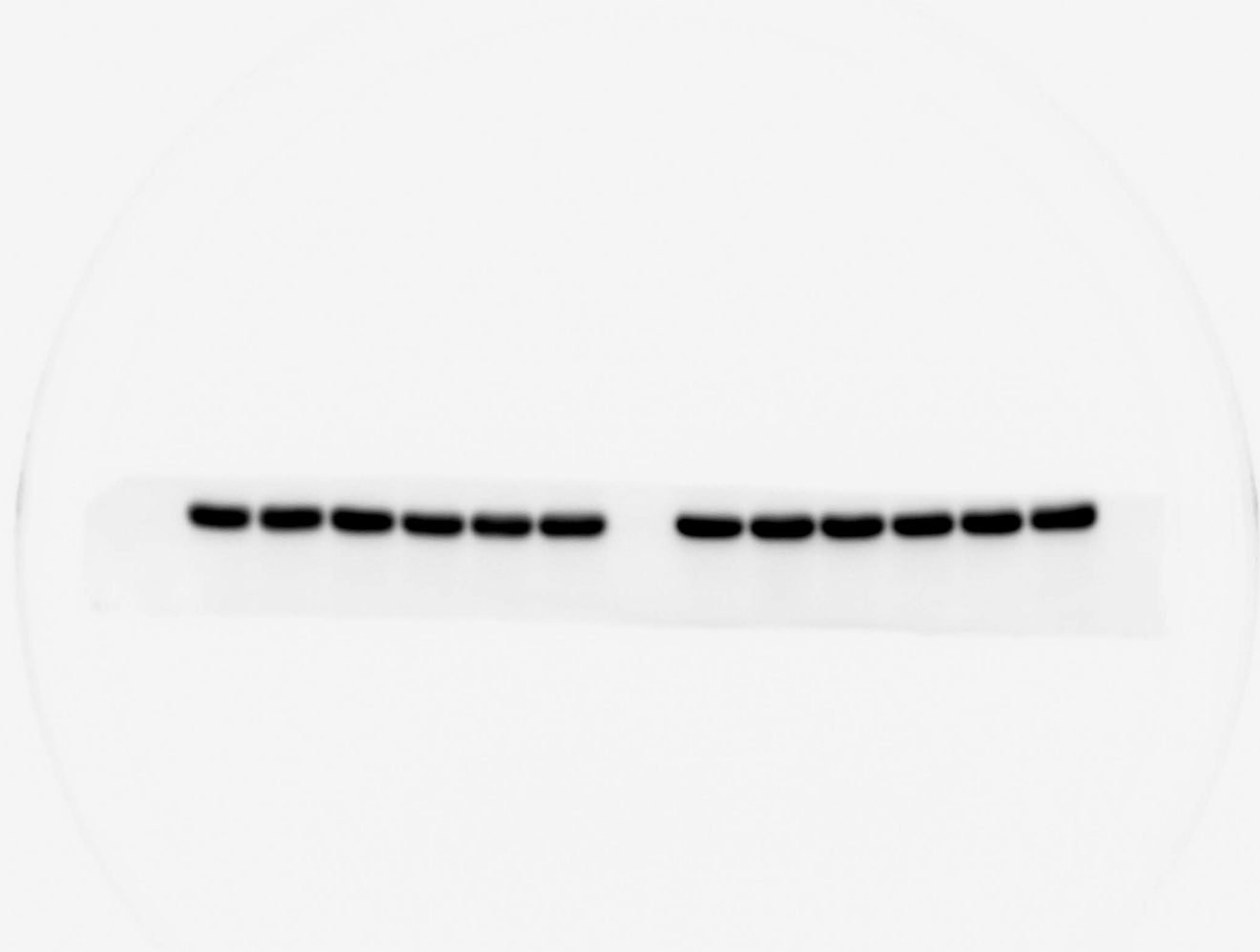

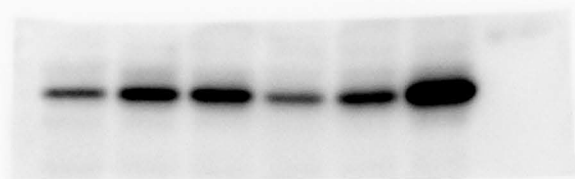

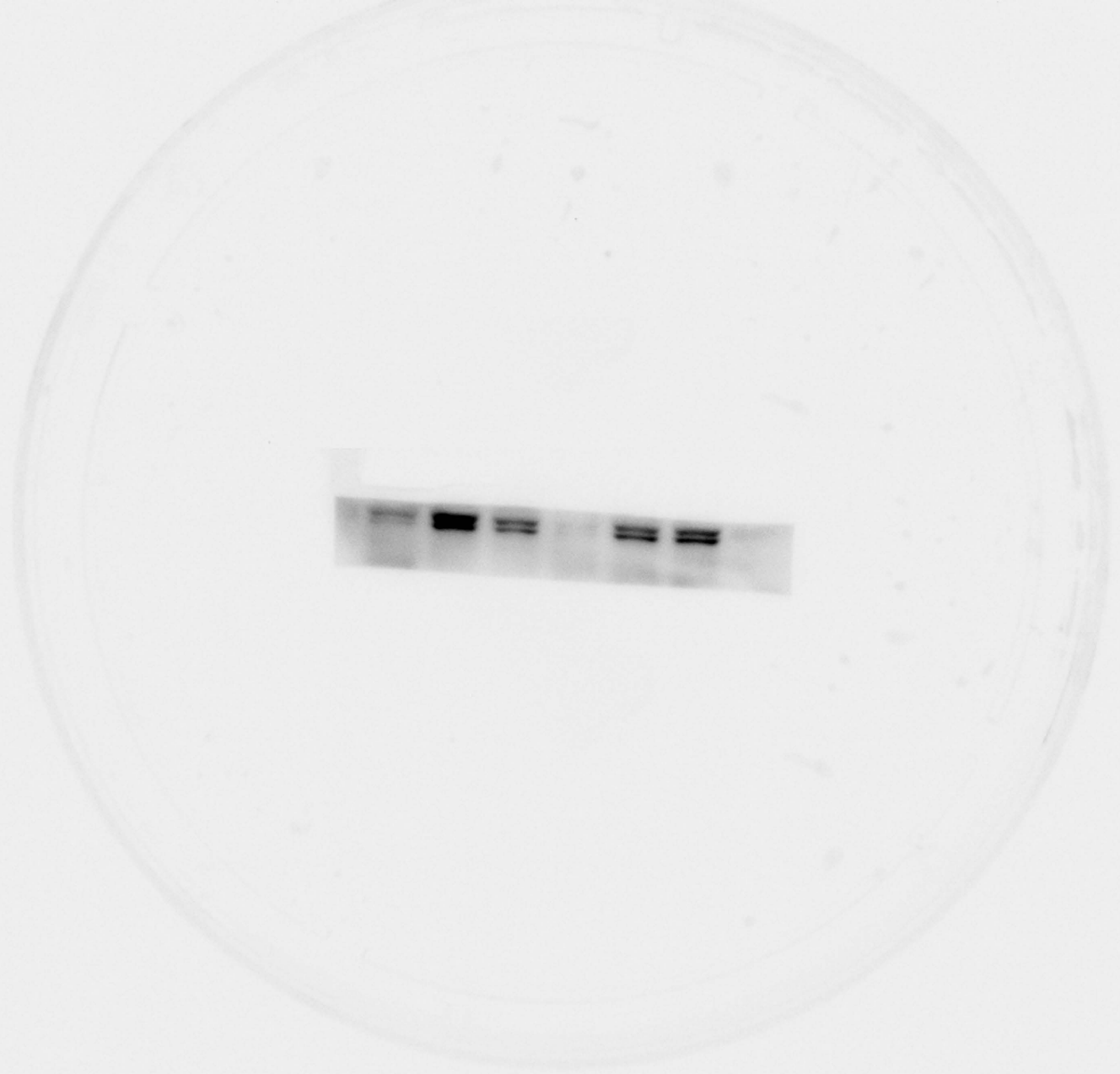

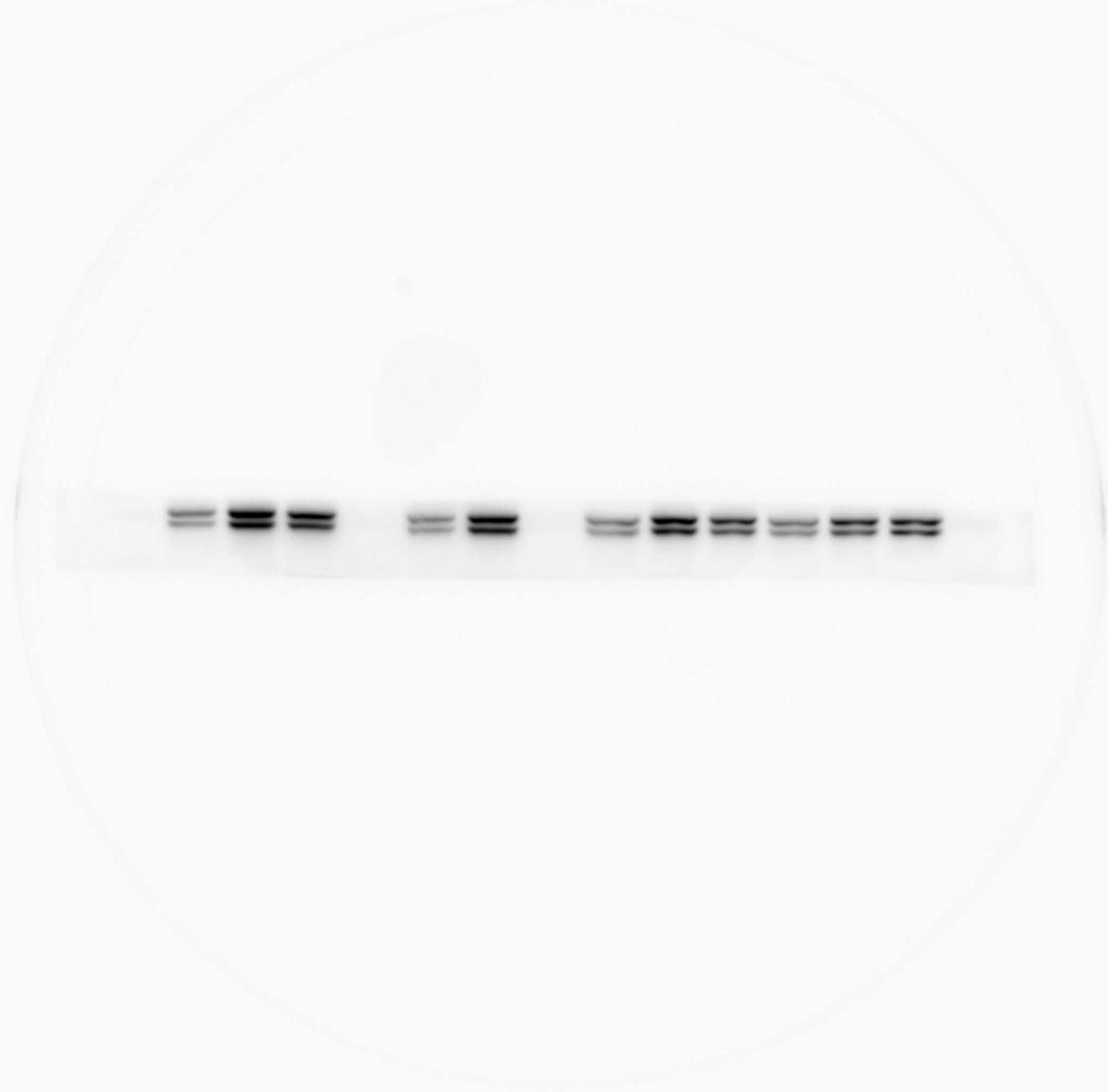

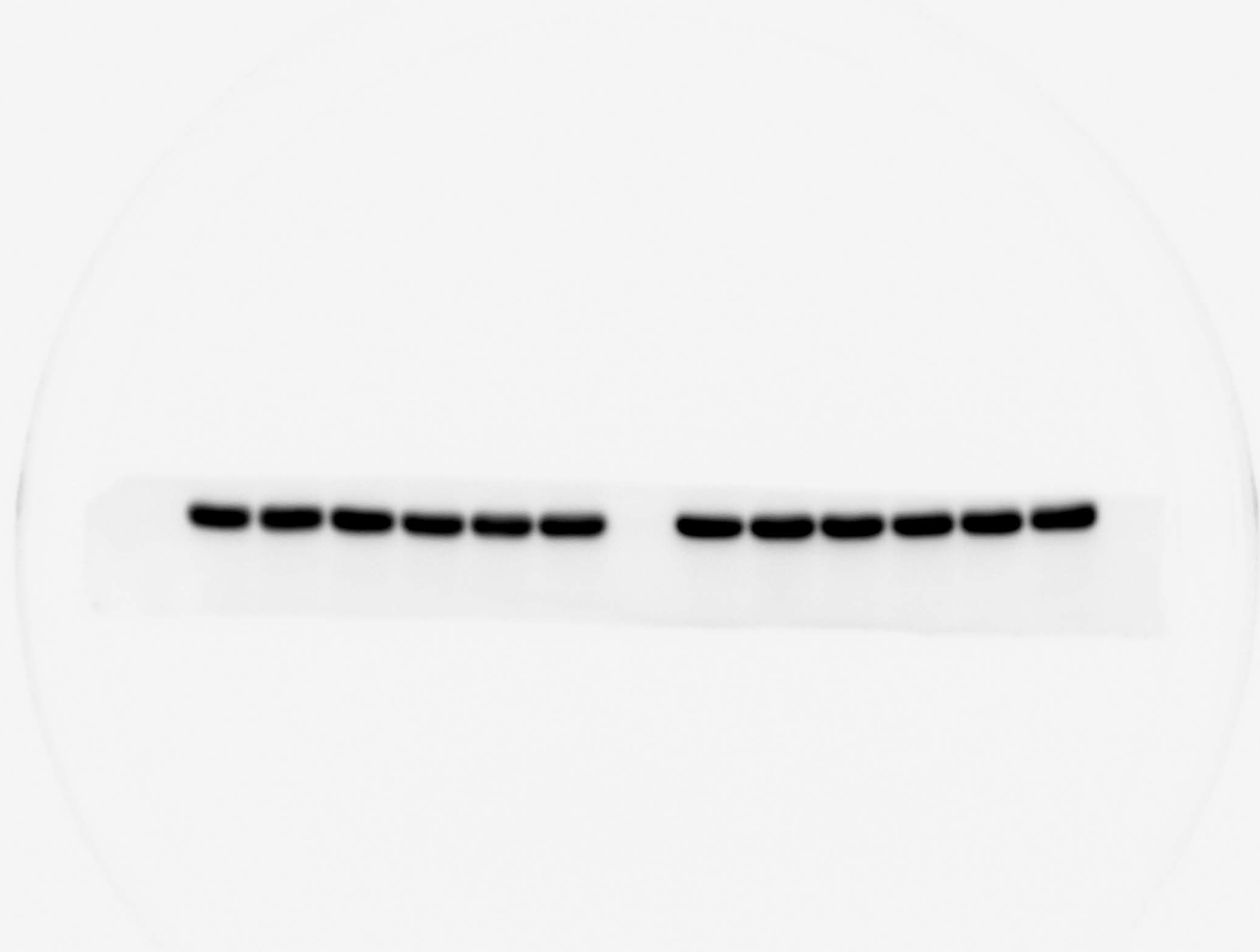

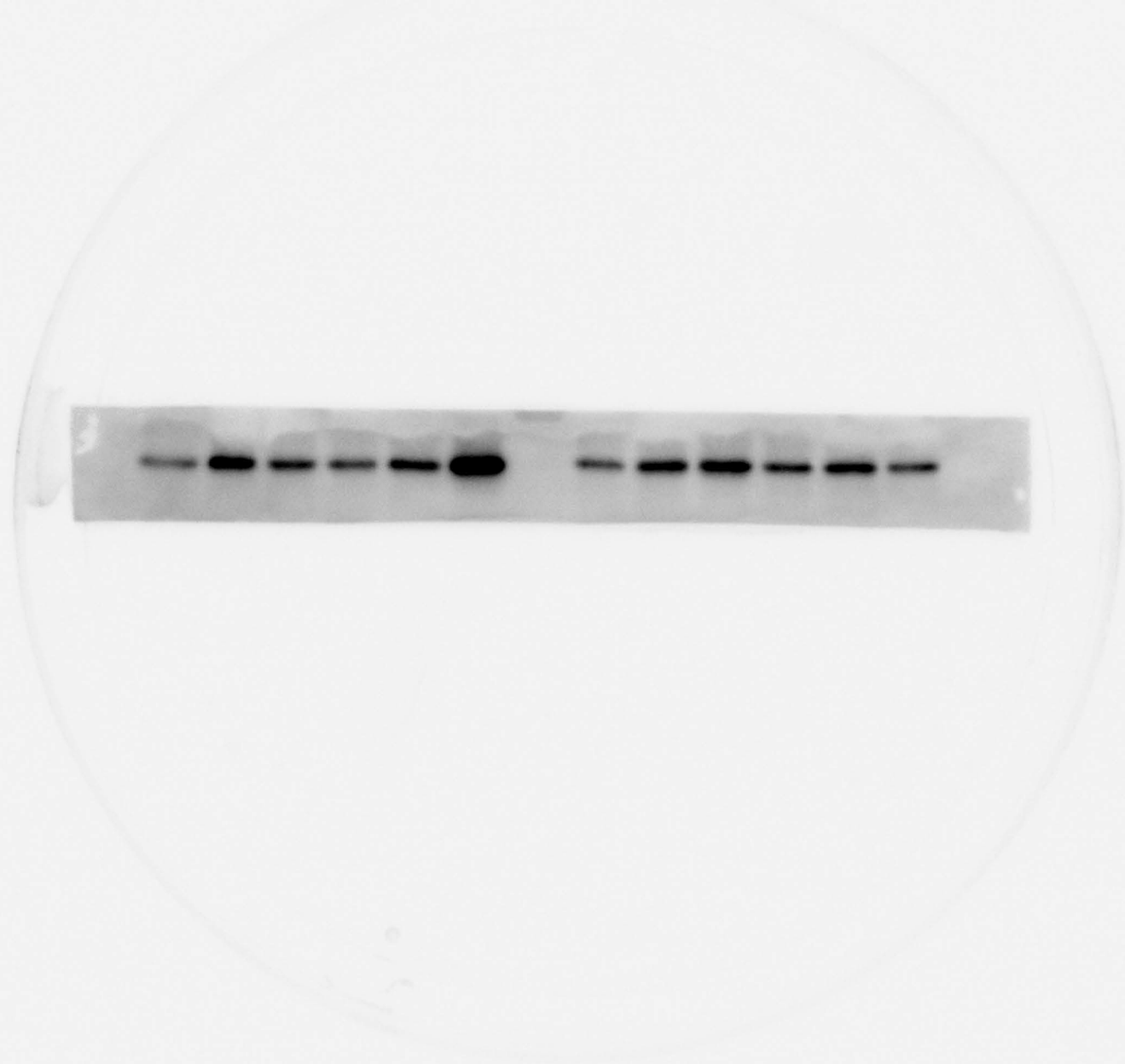

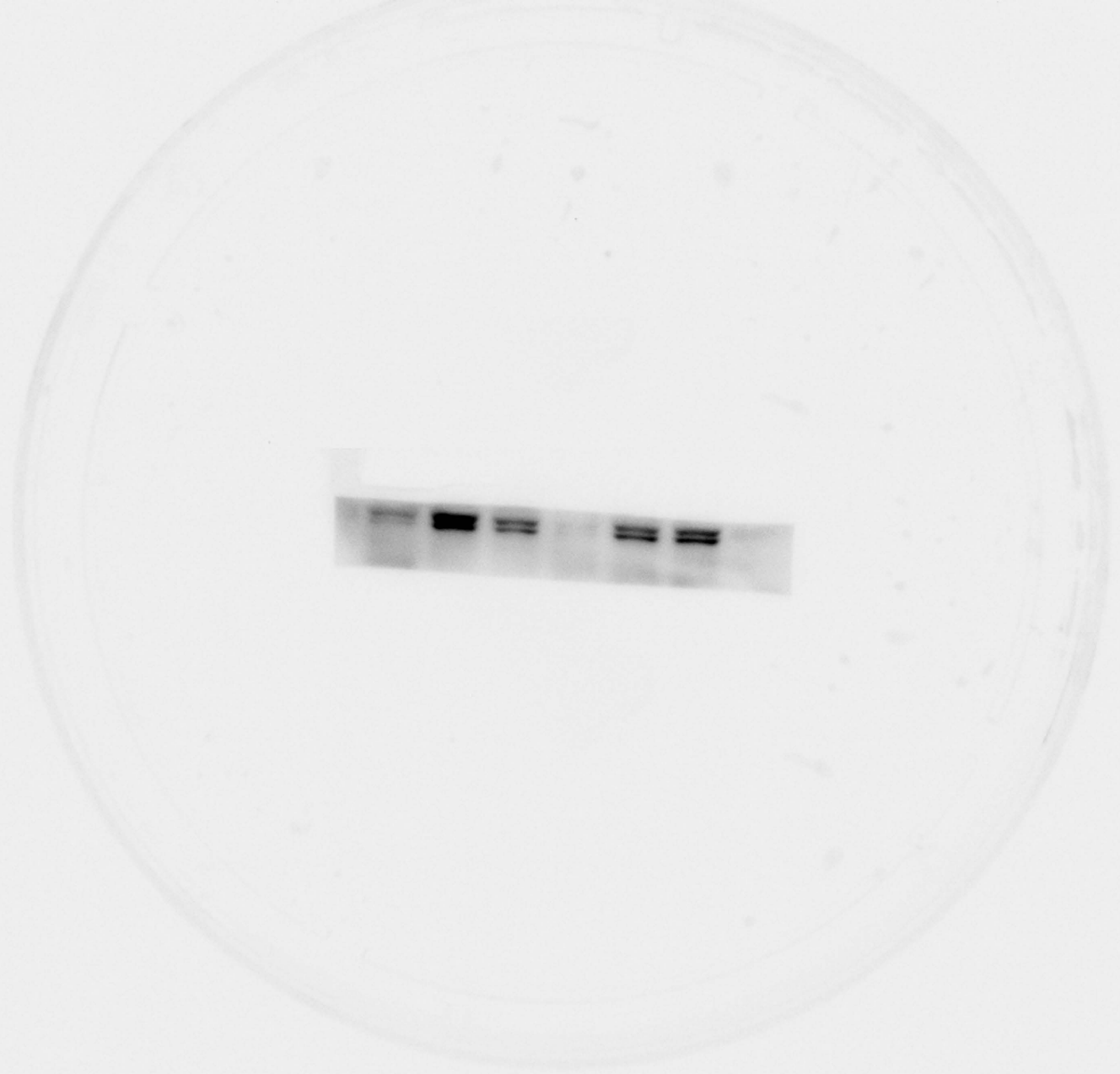

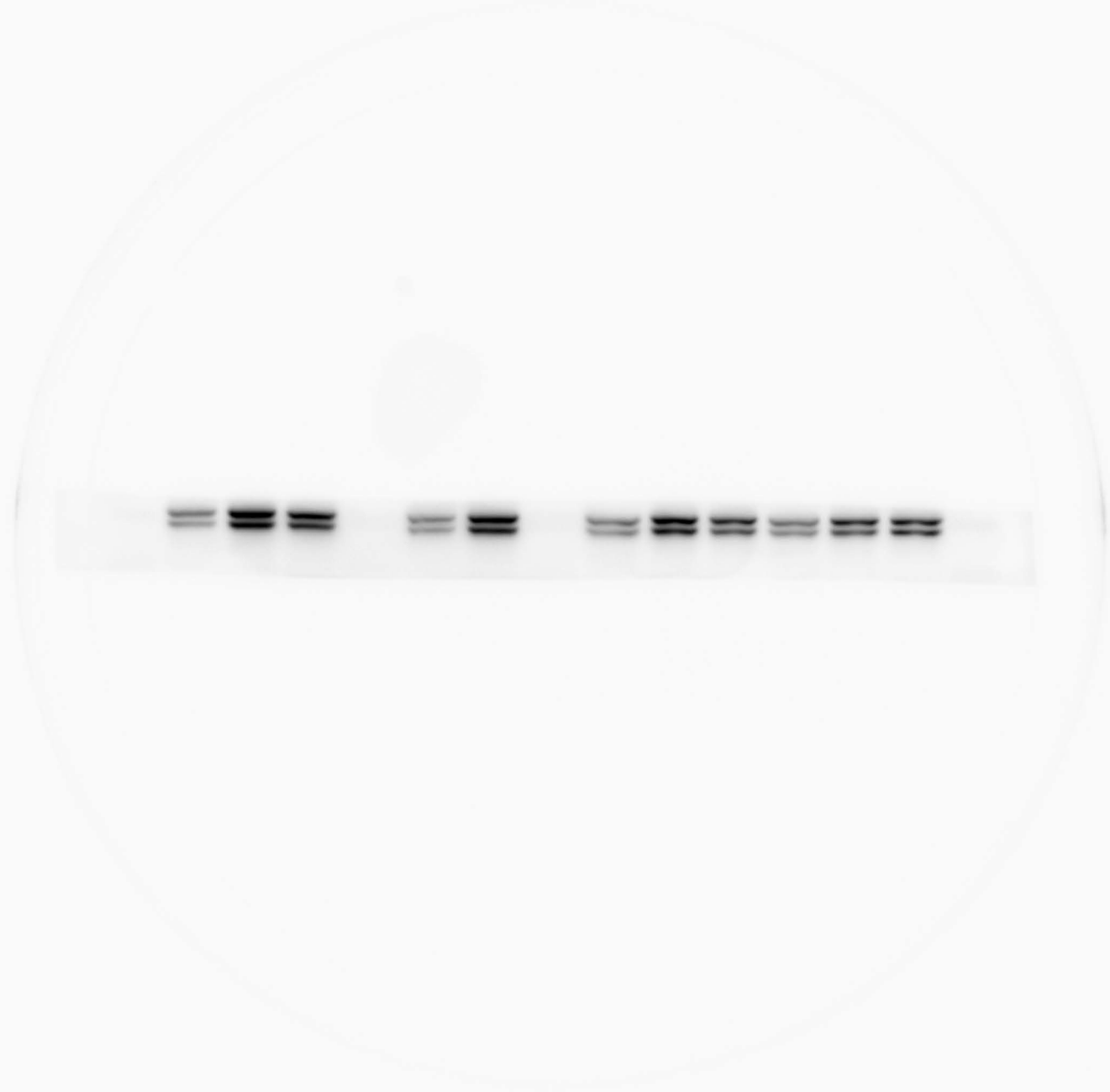

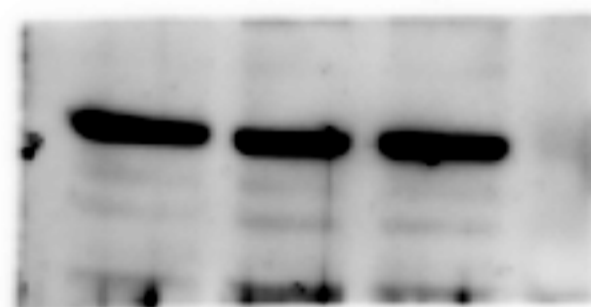

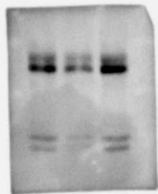

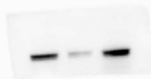

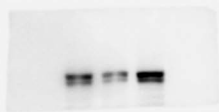

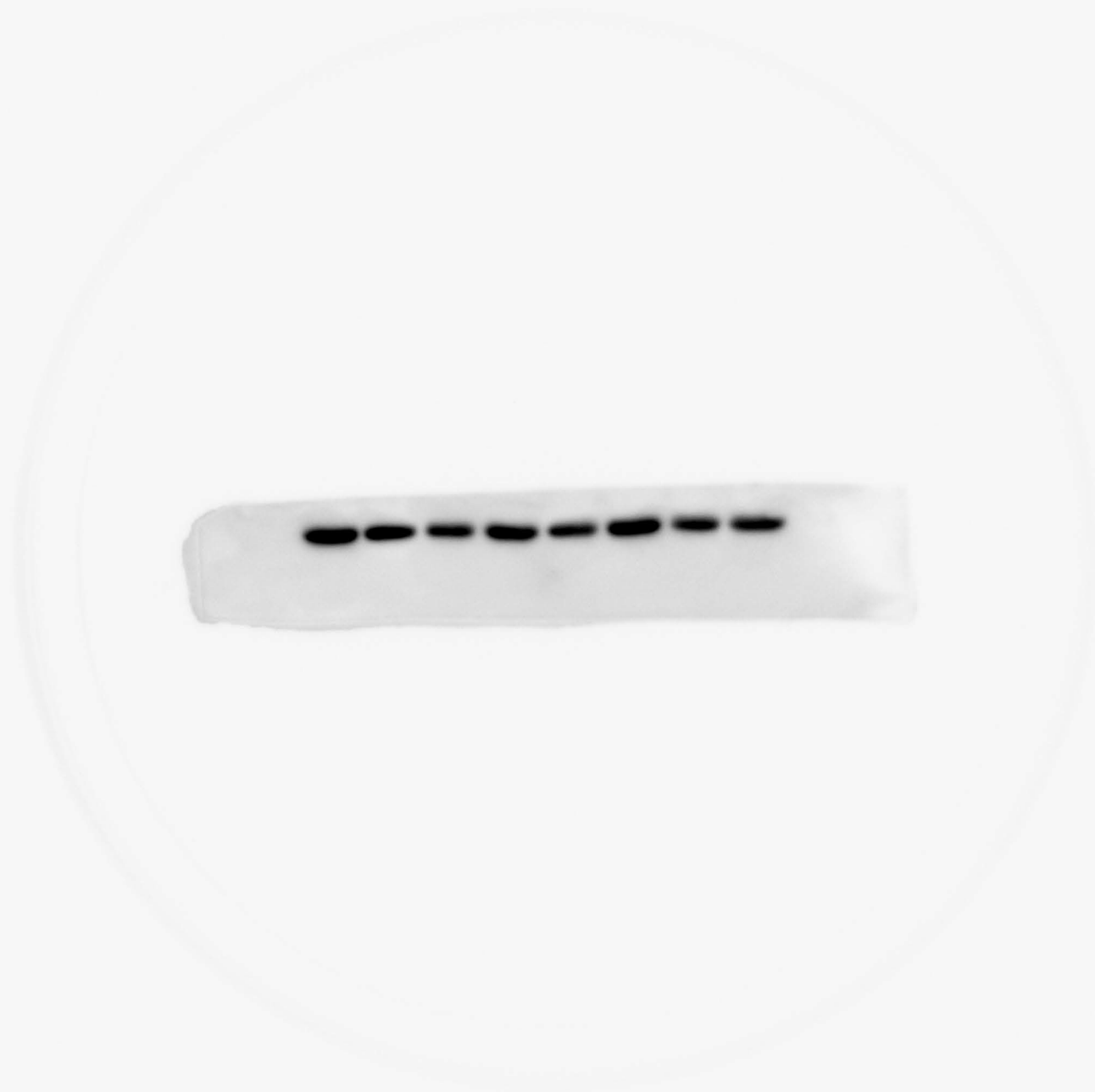

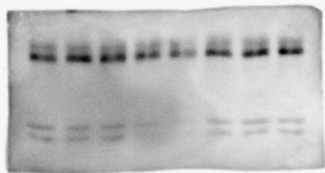

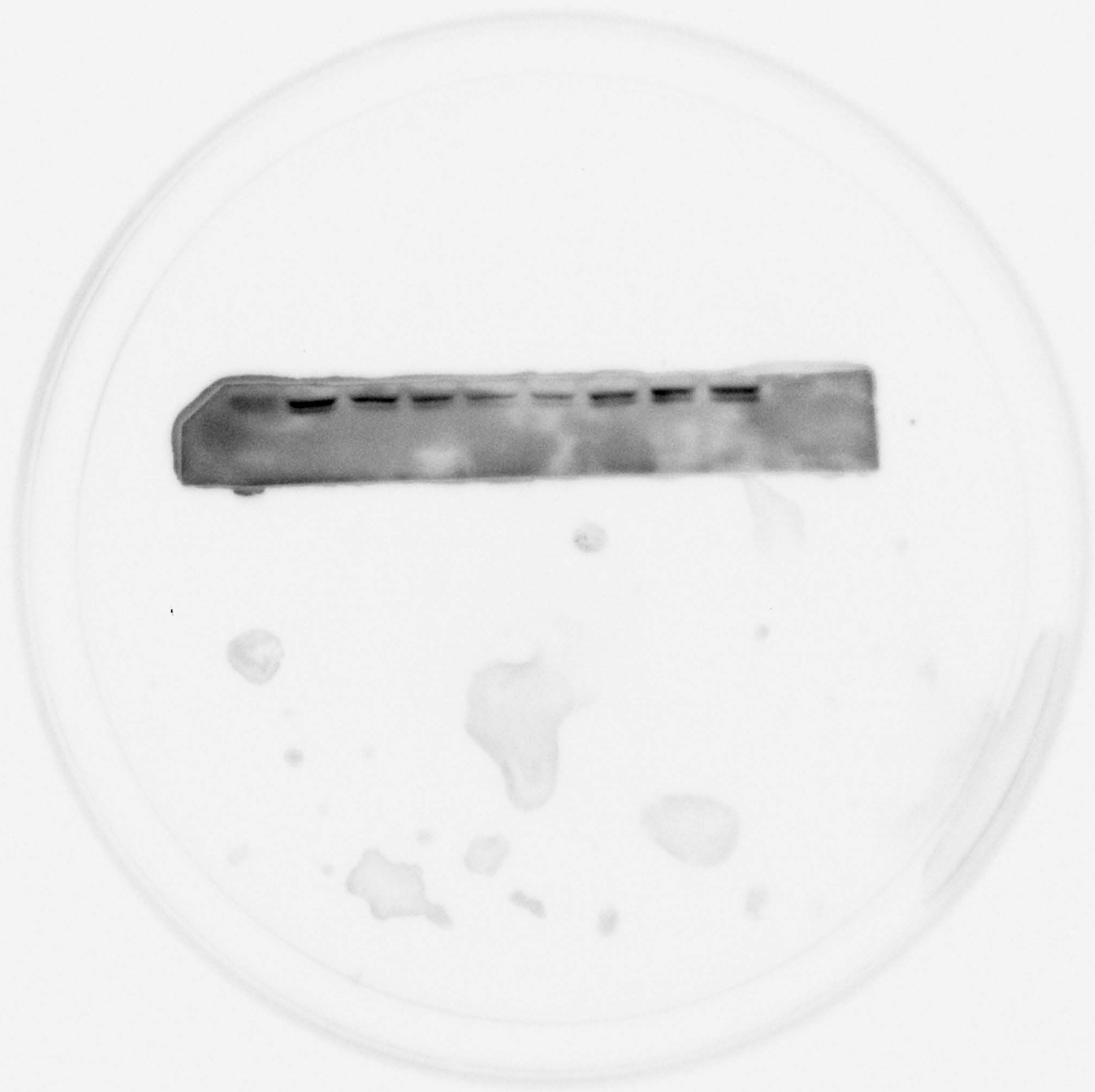

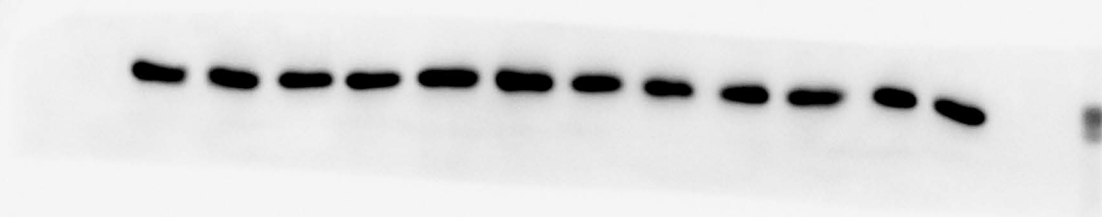

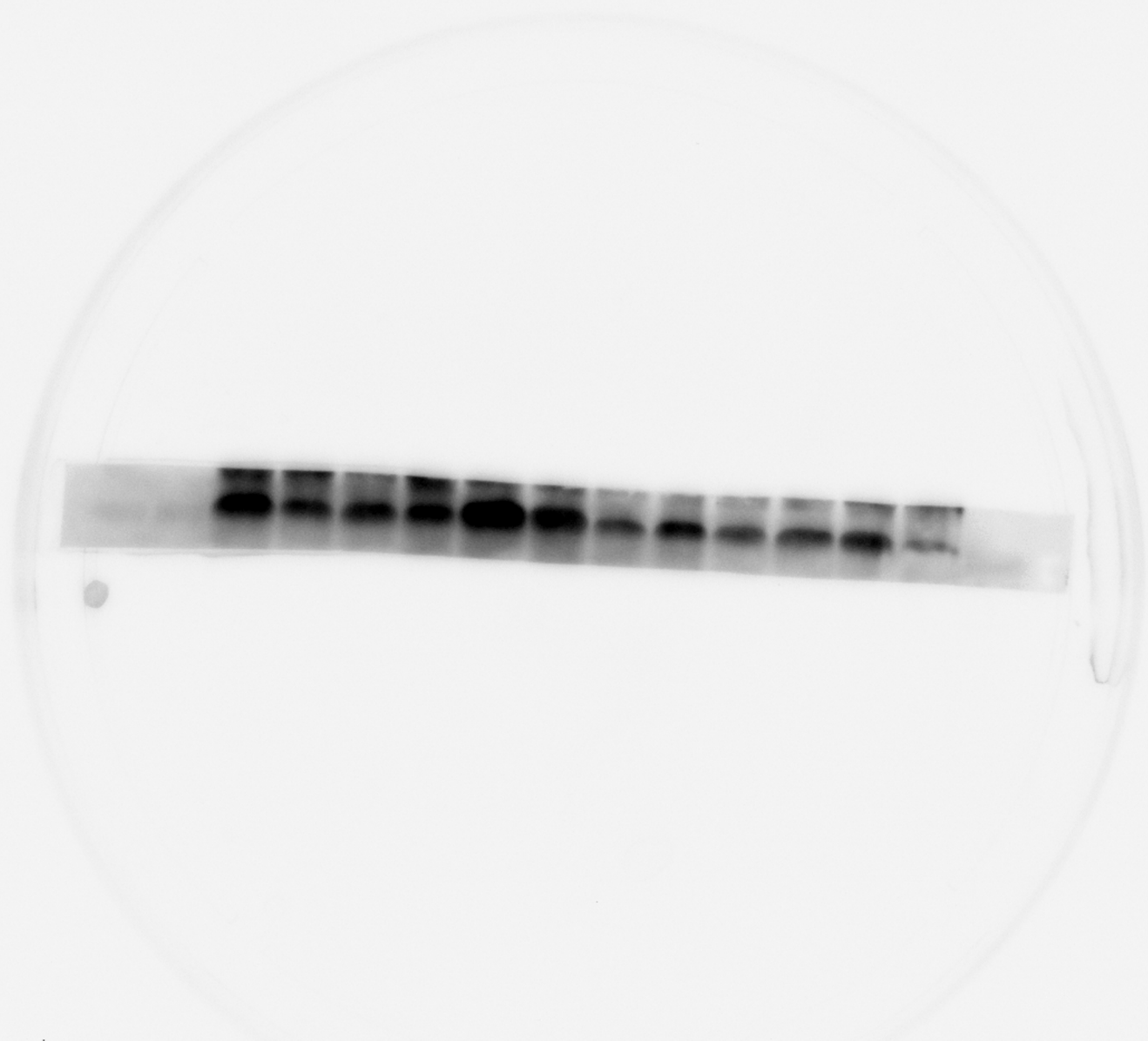

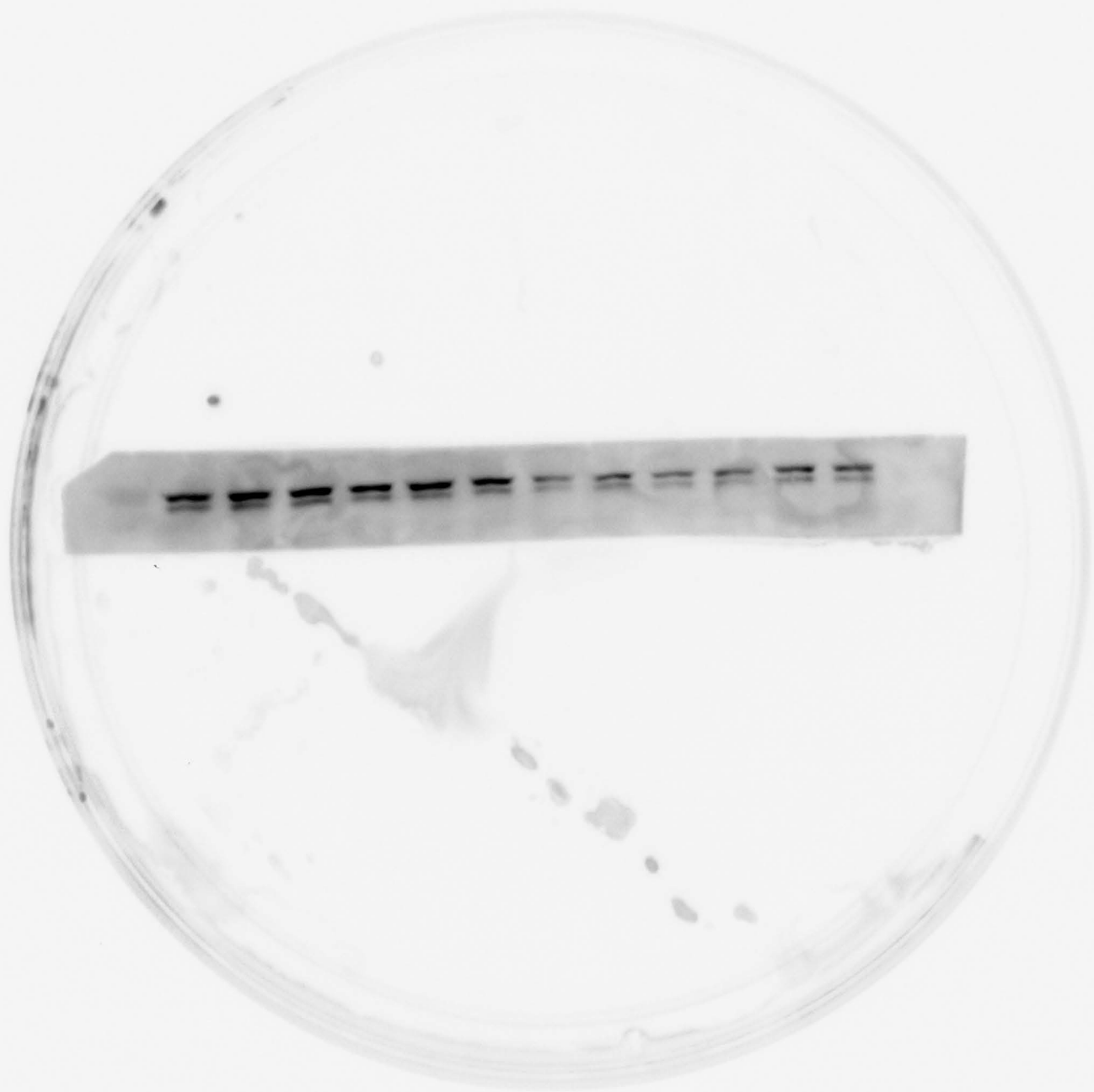

Supplement: Supplementary file 6 [file datasheet1.pdf]

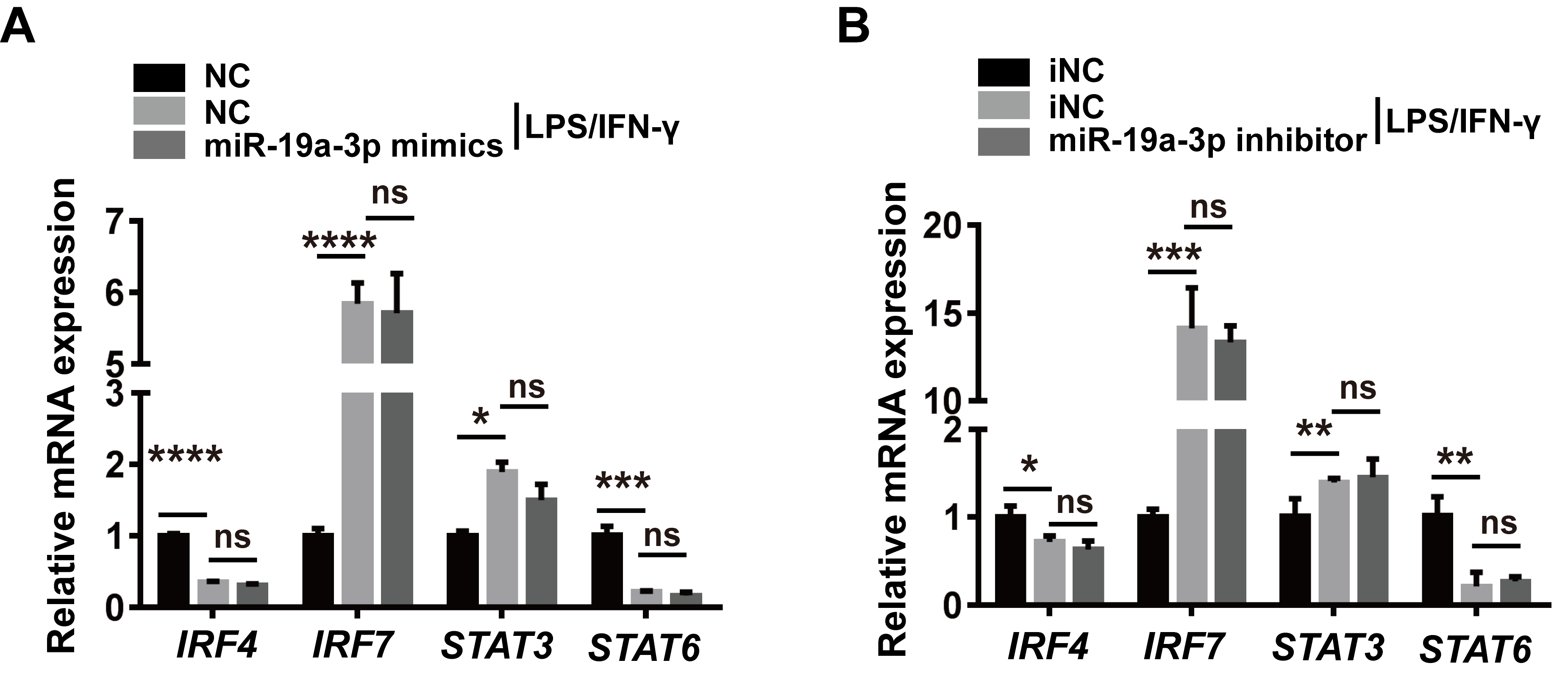

Supplement: Supplementary file 8 [file image5.tif]
