# Supplementary material for: MiR-19a-3p Suppresses M1 Macrophage Polarization by Inhibiting STAT1/IRF1 Pathway
Source: Front Pharmacol. 2021 May 4;12:614044. doi: 10.3389/fphar.2021.614044 (PMC8129022; doi:10.3389/fphar.2021.614044)
Supplement: Supplementary file 3 [file datasheet3.docx]

**Supplementary Figure legends**

**Supplementary Figure 1 Overexpressed miR-19a-3p suppresses BMDM derived M1 polarization and down-regulated miR-19a-3p improves this effect.**

BMDM cells transfected with miR-19a-3p mimics/NC or miR-19a-3p inhibitor/iNC for 24h, then stimulated with LPS plus IFN-γ for 24h. **(A, B)** miR-19a-3p was determined using qRT-PCR (data were pooled from three independent experiments). **(C, D)** *TNF-α, CXCL9, CXCL10, iNOS* were analyzed by qRT-PCR (data were pooled from three independent experiments). **(E, F)** Western blot analysis the protein level of iNOS (a representative blot, from three independent experiments). NC: miR-19a-3p mimics negative control, iNC: miR-19a-3p inhibitor negative control. Statistical significance was calculated using unpaired Student’s t-test or ANOVA followed by Bonferroni test. **P* < 0.05, ***P* < 0.01, ****P* < 0.001, *****P* < 0.0001.

**Supplementary Figure 2 The expression of M1 macrophage surface markers in RAW264.7 cells.**

(A)The expression of M1 macrophage markers CD80, CD86, MHCⅡ were detected by flow cytometry in RAW264.7 cells treated with LPS and IFN-γ for 24h after transfection of miR-19a-3p mimics/NC (a representative experiment, from three independent experiments). (B) The expression of M1 macrophage markers CD80, CD86, MHCⅡ were detected by flow cytometry in RAW264.7 cells treated with LPS and IFN-γ for 24h after transfection of miR-19a-3p inhibitor/iNC(a representative experiment, from three independent experiments). NC: miR-19a-3p mimics negative control, iNC: miR-19a-3p inhibitor negative control.

**Supplementary Figure 3 miR-19a-3p has no effect on the expression of** **arginase-1.**

RAW264.7 cells and BMDM cells transfected with miR-19a-3p mimics/NC or miR-19a-3p inhibitor/iNC for 24h, then stimulated with LPS plus IFN-γ for 24h. **(A, B)** *Arginase-1* were analyzed by qRT-PCR inRAW264.7 cells and BMDM derived macrophage (data were pooled from three independent experiments). **(C, D)** Western blot analysis the protein level of arginase-1 in RAW264.7 cells and BMDM derived macrophage (a representative blot, from three independent experiments). NC: miR-19a-3p mimics negative control, iNC: miR-19a-3p inhibitor negative control. Statistical significance was calculated using unpaired Student’s t-test or ANOVA followed by Bonferroni test. **P* < 0.05, ***P* < 0.01.

**Supplementary Figure 4** **miR-19a-3p inhibits the STAT1/IRF1 pathway in BMDM cells.**

BMDM cells were transfected with miR-19a-3p mimics/NC or miR-19a-3p inhibitor/iNC for 24h, then the cells were stimulated with LPS/IFN-γ for 24h. **(A, B)** *STAT1* and *IRF1* mRNA expression were detected by qRT-PCR (data were pooled from three independent experiments). **(C)** p-STAT1, STAT1, and IRF1 were measured by western blot (a representative blot, from three independent experiments). NC: miR-19a-3p mimics negative control, iNC: miR-19a-3p inhibitor negative control. Statistical significance was calculated using ANOVA followed by Bonferroni test. **P* < 0.05, ****P* < 0.001, *****P* < 0.0001.

**Supplementary Figure 5 miR-19a-3p has no effect on the expression of IRF4, IRF7, STAT3, STAT6.**

The mRNA level of *IRF4, IRF7, STAT3, STAT6* were detected in RAW264.7 cells transfected with miR-19a-3p mimics/NC or miR-19a-3p inhibitor/iNC for 24h, followed by stimulating with LPS/IFN-γ for 24h. NC: miR-19a-3p mimics negative control, iNC: miR-19a-3p inhibitor negative control. Statistical significance was calculated using ANOVA followed by Bonferroni test. **P* < 0.05, ****P* < 0.001, *****P* < 0.0001.
