# Supplementary material for: MiR-19a-3p Suppresses M1 Macrophage Polarization by Inhibiting STAT1/IRF1 Pathway
Source: Front Pharmacol. 2021 May 4;12:614044. doi: 10.3389/fphar.2021.614044 (PMC8129022; doi:10.3389/fphar.2021.614044)
Supplement: Supplementary file 7 [file datasheet2.docx]

**Figure 1C**

**iNOS**

The first line on the left is markers.

The fourth line on the left is the control group (RAW264.7).

The fifth line on the left is the LPS/IFN-γ treated RAW264.7 cells.

**p-STAT1**

The first line on the left and right are markers.

The fourth line on the left is the control group (RAW264.7).

The fifth line on the left is the LPS/IFN-γ treated RAW264.7 cells.

**STAT1**

The first line on the left and right are markers.

The fourth line on the left is the control group (RAW264.7).

The fifth line on the left is the LPS/IFN-γ treated RAW264.7 cells.

**GAPDH**

The third line on the left is the control group (RAW264.7).

The fourth line on the left is the LPS/IFN-γ treated RAW264.7 cells.

**Figure 3G**

**iNOS**

The third line on the right is RAW264.7 cells transfected with miR-19a-3p mimics negative control (NC) group.

The second line on the right is RAW264.7 cells transfected with NC, and then treated with LPS and IFN-γ group.

The first line on the right is RAW264.7 cells transfected with miR-19a-3p mimics, and then treated with LPS and IFN-γ group.

**GAPDH**

The third line on the right is RAW264.7 cells transfected with miR-19a-3p mimics negative control (NC) group.

The second line on the right is RAW264.7 cells transfected with NC, and then treated with LPS and IFN-γ group.

The first line on the right is RAW264.7 cells transfected with miR-19a-3p mimics, and then treated with LPS and IFN-γ group.

**Figure 3H**

**iNOS**

The third line on the right is RAW264.7 cells transfected with miR-19a-3p inhibitor negative control (iNC) group.

The second line on the right is RAW264.7 cells transfected with iNC, and then treated with LPS and IFN-γ group.

The first line on the right is RAW264.7 cells transfected with miR-19a-3p inhibitor, and then treated with LPS and IFN-γ group.

**GAPDH**

The third line on the right is RAW264.7 cells transfected with miR-19a-3p inhibitor negative control (iNC) group.

The second line on the right is RAW264.7 cells transfected with iNC, and then treated with LPS and IFN-γ group.

The first line on the right is RAW264.7 cells transfected with miR-19a-3p inhibitor, and then treated with LPS and IFN-γ group.

**Figure 4C Left (miR-19a-3p mimics group)**

**p-STAT1**

The first line on the left is RAW264.7 cells transfected with miR-19a-3p mimics negative control (NC) group.

The second line on the left is RAW264.7 cells transfected with NC, and then treated with LPS and IFN-γ group.

The third line on the left is RAW264.7 cells transfected with miR-19a-3p mimics, and then treated with LPS and IFN-γ group.

**STAT1**

The first line on the left is RAW264.7 cells transfected with miR-19a-3p mimics negative control (NC) group.

The second line on the left is RAW264.7 cells transfected with NC, and then treated with LPS and IFN-γ group.

The third line on the left is RAW264.7 cells transfected with miR-19a-3p mimics, and then treated with LPS and IFN-γ group.

**IRF1**

The third line on the right is RAW264.7 cells transfected with miR-19a-3p mimics negative control (NC) group.

The second line on the right is RAW264.7 cells transfected with NC, and then treated with LPS and IFN-γ group.

The first line on the right is RAW264.7 cells transfected with miR-19a-3p mimics, and then treated with LPS and IFN-γ group.

**GAPDH**

The first line on the left is RAW264.7 cells transfected with miR-19a-3p mimics negative control (NC) group.

The second line on the left is RAW264.7 cells transfected with NC, and then treated with LPS and IFN-γ group.

The third line on the left is RAW264.7 cells transfected with miR-19a-3p mimics, and then treated with LPS and IFN-γ group.

**Figure 4C Right (miR-19a-3p inhibitor group)**

**p-STAT1**

The fourth line on the left is RAW264.7 cells transfected with miR-19a-3p inhibitor negative control (iNC) group.

The fifth line on the left is RAW264.7 cells transfected with iNC, and then treated with LPS and IFN-γ group.

The sixth line on the left is RAW264.7 cells transfected with miR-19a-3p inhibitor, and then treated with LPS and IFN-γ group.

**STAT1**

The fourth line on the left is RAW264.7 cells transfected with miR-19a-3p inhibitor negative control (iNC) group.

The fifth line on the left is RAW264.7 cells transfected with iNC, and then treated with LPS and IFN-γ group.

The sixth line on the left is RAW264.7 cells transfected with miR-19a-3p inhibitor, and then treated with LPS and IFN-γ group.

**IRF1**

The third line on the right is RAW264.7 cells transfected with miR-19a-3p inhibitor negative control (iNC) group.

The second line on the right is RAW264.7 cells transfected with iNC, and then treated with LPS and IFN-γ group.

The first line on the right is RAW264.7 cells transfected with miR-19a-3p inhibitor, and then treated with LPS and IFN-γ group.

**GAPDH**

The fourth line on the left is RAW264.7 cells transfected with miR-19a-3p inhibitor negative control (iNC) group.

The fifth line on the left is RAW264.7 cells transfected with iNC, and then treated with LPS and IFN-γ group.

The sixth line on the left is RAW264.7 cells transfected with miR-19a-3p inhibitor, and then treated with LPS and IFN-γ group.

**Figure 5C**

**p-STAT1**

The first line on the left is co-transfected with mimics negative control (NC) and STAT1 vector plasmid and then treated with LPS and IFN-γ group.

The second line on the left is co-transfected with miR-19a-3p mimics and STAT1 vector plasmid and then treated with LPS and IFN-γ group.

The third line on the left is co-transfected with miR-19a-3p mimics and STAT1 overexpressed plasmid and then treated with LPS and IFN-γ group.

**STAT1**

The first line on the left is co-transfected with mimics negative control (NC) and STAT1 vector plasmid and then treated with LPS and IFN-γ group.

The second line on the left is co-transfected with miR-19a-3p mimics and STAT1 vector plasmid and then treated with LPS and IFN-γ group.

The third line on the left is co-transfected with miR-19a-3p mimics and STAT1 overexpressed plasmid and then treated with LPS and IFN-γ group.

**IRF1**

The first line on the left is co-transfected with mimics negative control (NC) and STAT1 vector plasmid and then treated with LPS and IFN-γ group.

The second line on the left is co-transfected with miR-19a-3p mimics and STAT1 vector plasmid and then treated with LPS and IFN-γ group.

The third line on the left is co-transfected with miR-19a-3p mimics and STAT1 overexpressed plasmid and then treated with LPS and IFN-γ group.

**GAPDH**

The first line on the left is co-transfected with mimics negative control (NC) and STAT1 vector plasmid and then treated with LPS and IFN-γ group.

The second line on the left is co-transfected with miR-19a-3p mimics and STAT1 vector plasmid and then treated with LPS and IFN-γ group.

The third line on the left is co-transfected with miR-19a-3p mimics and STAT1 overexpressed plasmid and then treated with LPS and IFN-γ group.

**Figure 7F**

**IRF1**

The third to fifth lines on the left are control mice group.

The sixth to the eighth lines on the left are the mice group that challenged with LPS.

**STAT1**

The first line on the left is marker.

The fourth to sixth lines on the left are control mice group.

The seventh to the ninth lines on the left are the mice group that challenged with LPS.

**GAPDH**

The third to fifth lines on the left are control mice group.

The sixth to the eighth lines on the left are the mice group that challenged with LPS.

**Figure 8F**

**IRF1**

The third to eighth lines on the left are agomiR NC group challenged with LPS.

The nineth to the fourteenth on the left are the agomiR-19a-3p group challenged with LPS.

**STAT1**

The first line on the left is marker.

The second to seventh lines on the left are agomiR NC group challenged with LPS.

The eighth to the thirteenth on the left are the agomiR-19a-3p group challenged with LPS.

**GAPDH**

The first to sixth lines on the left are agomiR NC group challenged with LPS.

The seventh to the twelfth on the left are the agomiR-19a-3p group challenged with LPS.
